# Synthesis, Crystal Design and Anticancer Potential of Novel Cu(II), Ni(II), and Pd(II) Complexes with Carbazate Ligand

**DOI:** 10.1021/acsomega.5c02365

**Published:** 2025-05-22

**Authors:** Daniel J. de Siqueira, Mariana P. Viana, Katia M. Oliveira, Claudia C. Gatto

**Affiliations:** Institute of Chemistry, Laboratory of Inorganic Synthesis and Crystallography, 28127 University of Brasilia, Asa Norte, Federal District, Brasília 70904-970, Brazil

## Abstract

The current study reports the synthesis and characterization of the ligand 2-acetylpyridine-methylcarbazate (Hapmc), and its metal complexes [Cu­(apmc)­Cl]_2_ (1), [Cu­(Hapmc)­Br_2_] (2), [Ni­(apmc)_2_] (3) and [Pd­(apmc)­Cl]_2_ (4). All compounds were characterized by single-crystal X-ray diffraction and spectroscopic analyses. The free carbazate ligand is in the keto tautomer and coordinates in this way to form complexes (2) and (4) and is converted to its enolic tautomer to form complexes (1) and (3). The crystal structures of complexes (1) and (2) revealed a dimer and a monomer compound, respectively, with a distorted square pyramid geometry with each metal center coordinated to a ligand molecule by the *NNO* system and two halide ions (Cl^–^ or Br^–^). Complex (3) presents an octahedral geometry with the Ni­(II) atom coordinated to two deprotonated ligand molecules. In contrast, the dimer (4) presents an unusual coordination mode by *NNN*-donor atoms of the ligand and each Pd­(II) has a square planar geometry. Noncovalent interactions were evaluated in qualitative and quantitative studies by computational calculations of the Hirshfeld surfaces and showed the most contribution to the formation of the crystal lattice are H···H interactions. The synthesized compounds were investigated in vitro against human cancer cells of A549 lung cancer, MCF-7 breast cancer, A2780cis cisplatin-resistant ovarian cancer, and noncancerous human lung cell MRC-5. All metal complexes exhibit better anticancer activity when compared to the free ligand and, in some cases, outperform the widely used reference drugs, showing strong potential in cancer treatment.

## Graphical abstract

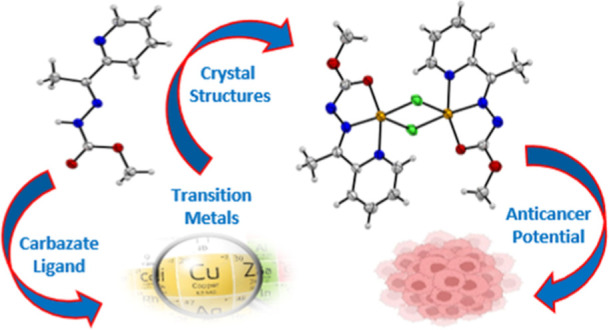

## 1 Introduction

The search for better drugs that are more efficient and have fewer side effects is essential in treating several diseases. In this context, Schiff bases emerge as promising candidates due to their anticancer, antibacterial, and antifungal properties. When coordinated to a metal center, Schiff bases have modified electronic properties, which can increase their biological activities.^1,2^ Studies have shown that many metal complexes with Schiff base ligands have interesting and promising biological applications.^3–5^ Carbazates are a class of Schiff bases obtained through a condensation reaction between an aldehyde or ketone and a carbazate derivative. Although carbazates have received less attention in the literature than other Schiff bases, studies with carbazate ligands have shown promising pharmacological results with potential antimicrobial and anticancer activities.^6–9^

In addition, carbazate compounds are used as intermediates in organic synthesis, medicinal chemistry, and other fields.^10–13^ Some transition metal complexes using carbazate as a ligand have been reported.^6,9,14^ The ability to coordinate with transition metals due to the variety of potential oxygen and nitrogen donor atoms can produce stable metal complexes with these Schiff bases, and these metal complexes can exhibit enhanced biological activity when compared with the free ligands. This behavior has generated particular interest in complexes containing ions such as Co­(II), Ni­(II), Pd­(II), and Pt­(II), attracting the attention of researchers due to their thermal, antibacterial, and antitumor properties.^11,15^

Copper complexes have received great attention due to their ability to fight diseases such as cancer and their capacity to inhibit DNA synthesis.^16^ Other transition metals, such as palladium and nickel, with similar properties to platinum but with less nephrotoxicity, are good candidates for possible new drugs.^17^ Several palladium complexes have been evaluated as anticancer agent due to their similarity to platinum complexes. Studies indicate that the biological activity is related to the ligands coordinated with the metal center.^17,18^ Nickel, which also belongs to the same group as platinum and palladium, plays a role in some biological processes, such as iron absorption and the metabolism of glucose and adrenaline. Due to the natural presence of nickel in human physiology, the human body already possesses mechanisms to regulate nickel concentration.^19^ Both palladium and nickel complexes have promising biological properties and may exhibit better biological activity when compared to the free ligand.^20–24^

This work describes the synthesis and design of the ligand Hapmc (2-acetylpyridine-methylcarbazate) and its metal complexes of Cu­(II), Ni­(II), and Pd­(II). All the synthesized compounds were characterized by single-crystal X-ray diffraction, FT-IR, electronic spectroscopy, mass spectrometry, and elemental analyses. The ligand Hapmc was also characterized by ^1^H NMR and ^13^C NMR techniques. The synthesized compounds had their biological activities evaluated against human cancer cells of A549 lung cancer, MCF-7 breast cancer, A2780cis cisplatin-resistant ovarian cancer, and noncancerous human lung cell MRC-5.

## 2 Results and Discussion

A condensation reaction between an equimolar amount of methylcarbazate and 2-acetylpyridine in ethanol and reflux synthesized the Schiff base 2-acetylpyridine-methylcarbazate (**Hapmc**). The complexation reactions of **Hapmc** with CuCl_2,_ CuBr_2_, NiCl_2,_ and PdCl_2_ yielded four new complexes [Cu­(apmc)­Cl]_2_ **(1)**, [Cu­(Hapmc)­Br_2_] **(2)**, [Ni­(apmc)_2_] **(3),** and [Pd­(apmc)­Cl]_2_ **(4)**, as represented in Figure 1. The compounds were characterized by single-crystal X-ray diffraction, physicochemical, and spectroscopy analysis.

**Figure 1:**
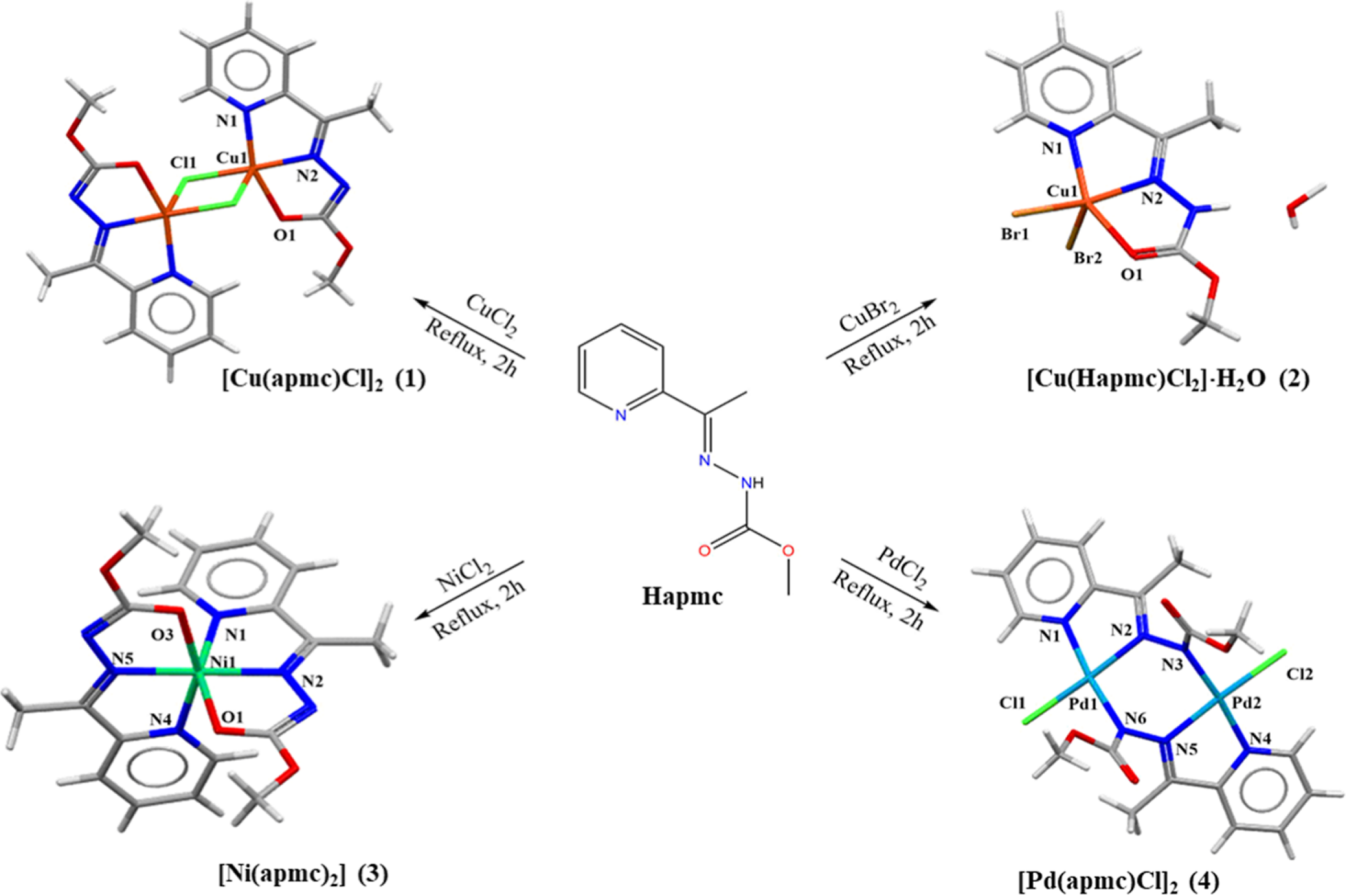
Synthesis of the Cu­(II), Ni­(II) and Pd­(II) complexes **(1–4)**.

### 2.1 Structural Analyses

The crystal structure of **Hapmc** and its metal complexes **(1–4)** was elucidated by single-crystal X-ray diffraction. This ligand can present keto–enol tautomerism in the −HN–C=O functional group, and the X-ray analysis revealed the free ligand in the keto form and *E* isomer, Figure 2a. The IR spectrum shows the ν­(C=O) band around 1705 cm^–1^, and the ^1^H NMR spectrum does not show any peak attributable to the O–H proton, suggesting the keto tautomer in the solid state and solution. The bond length C8–O1 of 1.213(2) Å is characteristic of a double bond, and the bond length C8–N3 of 1.348(2) Å is characteristic of a single bond. Similar behavior is observed in the reported works.^8,11,25^ The ligand **Hapmc** also exhibits intermolecular hydrogen bonds between N3–H3A···O1 with a bond length of 2.07 Å, Figure 2b.

**Figure 2:**
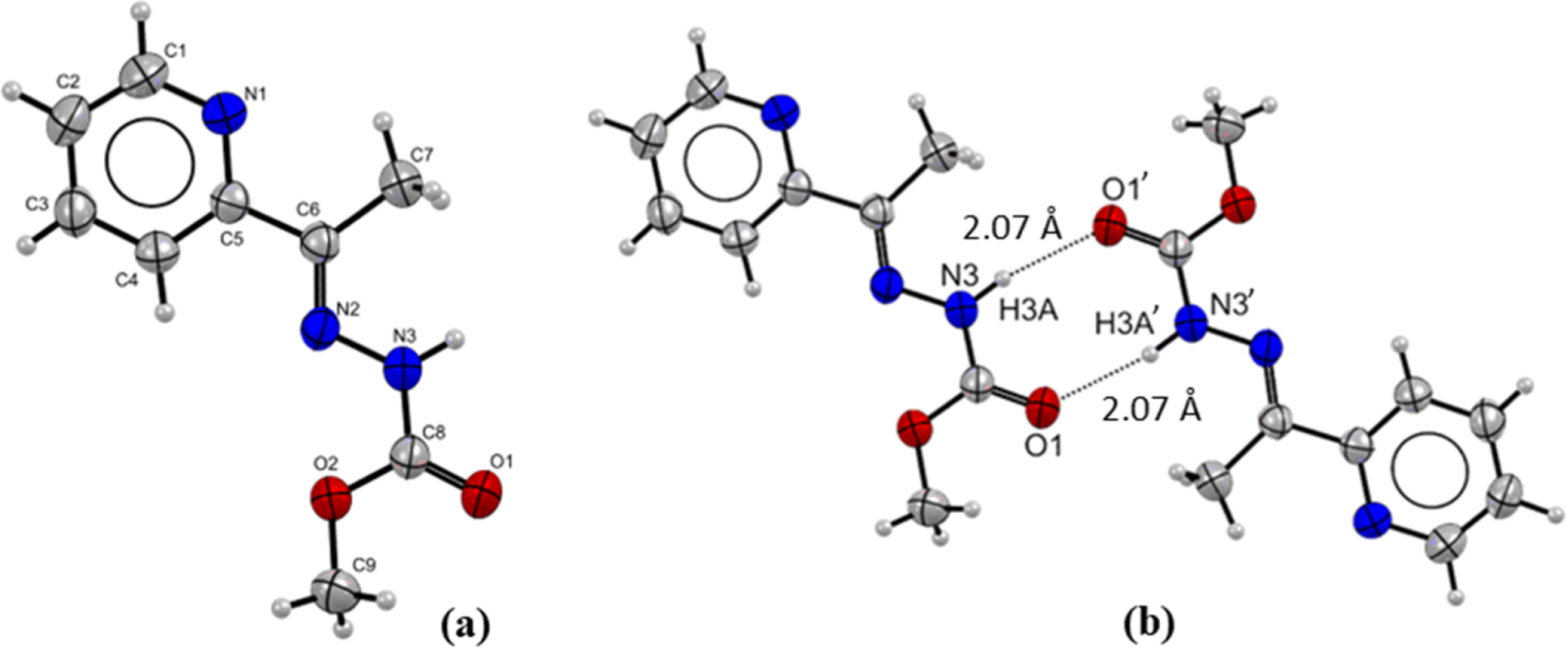
(a) Molecular structure of **Hampc** with crystallographic labeling (thermal ellipsoids with 30% probability). (b) Projection view of **Hampc** with hydrogen bonds as dashed lines (symmetry code ’–*x*, −*y* + 1, −*z* + 2).

The crystal structure determination of **(1)** revealed a dimer centrosymmetric copper­(II) complex with deprotonated ligand molecules coordinated by an *NNO*-donor system, Figure 3a. The metal centers are pentacoordinated with square pyramidal geometry, and two chloride bridges are located between the two copper­(II) atoms. The ligand is coordinated in the enol tautomer, as evidenced by the increased bond lengths C8–O1 of 1.265(6) Å compared with the bond lengths of 1.213(2) Å in the free ligand. It is observed that decreased bond lengths C8–N3 of 1.338(6) Å in **(1)** compared to 1.348(2) Å in **Hapmc**, which is characteristic of a double bond between carbon and nitrogen atoms.^9,14,26^ Any interaction between the metal ions is observed due to the long distance Cu–Cu’ of 3.434(2) Å.

**Figure 3:**
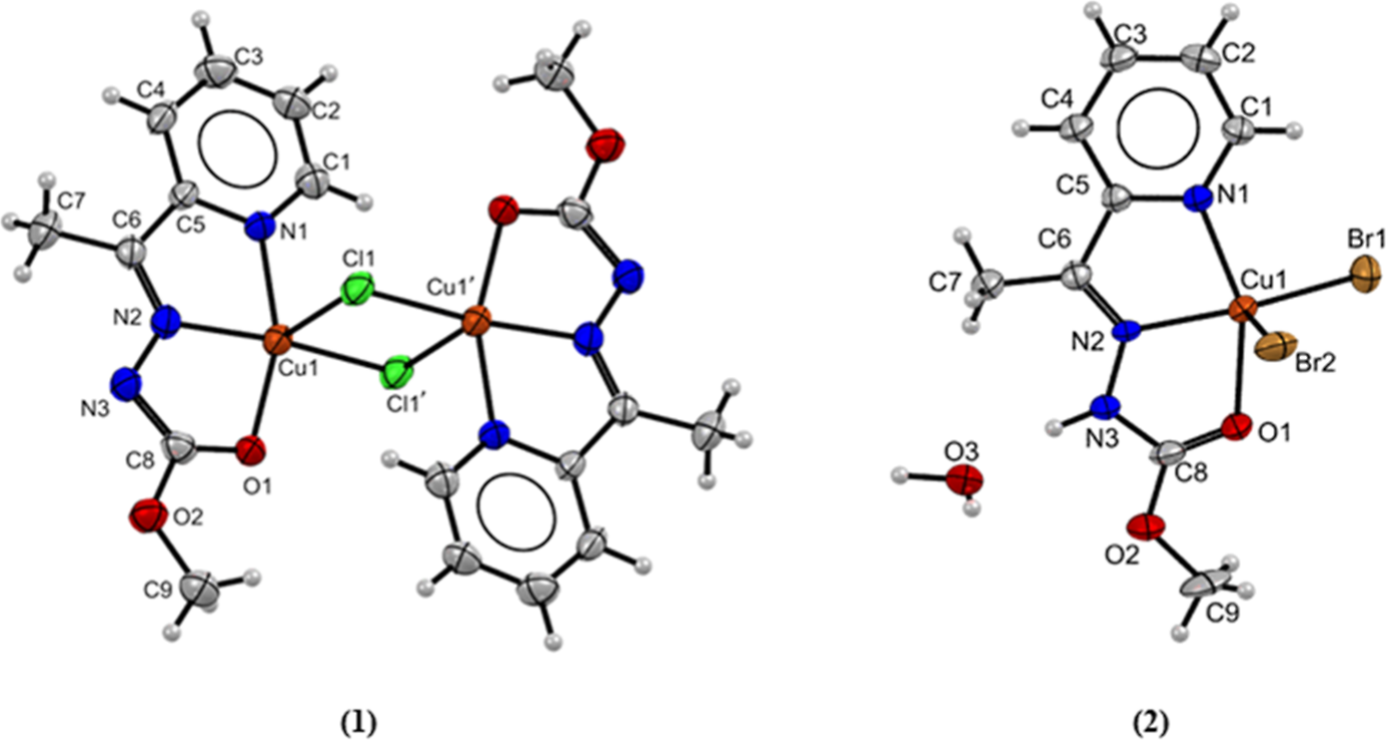
Molecular structure of **(1)** and **(2)** with crystallographic labeling. Thermal ellipsoids with 30% probability. Symmetry code ‘–*x* + 3/2, −*y* + 3/2, −*z* + 1.

In complex **(2)**, the copper atom is coordinated with two bromide ions and a tridentate ligand molecule with a square pyramidal geometry, Figure 3b. One water solvent molecule is also present in the unit cell. In contrast to the complex **(1)**, the complex **(2)** presents the ligand molecule in keto tautomer. The bond distances C8–O1 and C8–N3 are 1.232(6) and 1.355(6) Å, respectively. Intermolecular hydrogen bonds were observed between the water solvent molecule with a bromide ion and the nitrogen atom, and distances of 2.329 and 2.885 Å, respectively, and these bond length values are similar to those reported in the literature.^14,26^

The Addison parameter (τ_5_) was used to determine the geometry of pentacoordinated compounds quantitatively.^27^ The expression calculates this parameter: $\tau_{5}=\frac{\beta -\alpha }{60}$ , where α and β are the greatest angles of the coordination polyhedron, with β > α. Values close to 1 indicate trigonal bipyramidal geometries, and values close to 0 indicate square pyramidal geometries. The value of τ_5_ was calculated with the angles N2–Cu1–Cl1 of 169.95(2)° and N1–Cu1–O1 of 159.03(2)° for **(1)**, and N2–Cu1–Br1 of 155.85(2)° and N1–Cu1–O1 of 153.92(2)° for **(2)**. The Addison parameter found 0.182 and 0.032 for complexes **(1)** and **(2)**, respectively, indicating a distorted square pyramidal geometry. In both complexes, the apical positions are occupied by one halogen ion, and the ligand atoms and the second halogen ion present in the structures form the base of the pyramid. In **(1)**, the observed bond distance Cu1–Cl1 at the base of the pyramid of 2.223(2) Å is shorter than the bond length Cu1–Cl1’ at the apical position of 2.743(3) Å.^28–30^ The four basal atoms N1, N2, O1, and Cl1 are nearly coplanar, and the metal center is displaced by 0.166 Å from this plane toward the apical ligand. The *cis* angles on the Cu­(II) atoms range from 79.17(2) to 100.00(2)°, and the trans angles are 159.03(2) and 169.85(2)°. In the case of complex **(2)**, the bromide ion at the apical position also exhibited a longer bond length of 2.578(9) Å than the bond distance Cu1–Br1 of 2.358(8) Å at the base of the pyramid.^25,31^ The Cu­(II) ion is displaced by 0.371 Å from the basal plane composed of the N1, N2, O1, and Br1 atoms. The observed cis angles on the metal center range from 77.73(2) to 99.10(2)°, and the trans angles are 153.92(2) and 155.85(2)°.

Complex **(3)** was synthesized using the ligand **Hapmc** and NiCl_2_ in a 2:1 ratio (Figure 4). Two deprotonated ligand molecules in the enol form coordinate the metal center through the *NNO* donor system. The metal center is coordinated by two nitrogen atoms of the pyridine rings, two azomethine nitrogen atoms, and two oxygen atoms of the carbazate portion of the ligand. The coordination number for the Ni­(II) atom is six and distortion of the octahedral geometry is observed with the cis bond angles of the coordination sphere of the complex presenting values in the range of 76.69(8) and 103.25(2)° and the trans angles between 155.45(8) and 177.21(2)°, which are respectively different from the ideal angles of 90° and 180° for this geometry. The ligand is coordinated in the enol form with bond lengths C8–O1 and C17–O3 of 1.247(3) Å and 1.240(3) Å, respectively, corresponding to the values of a single bond between oxygen and carbon atoms.^25^

**Figure 4:**
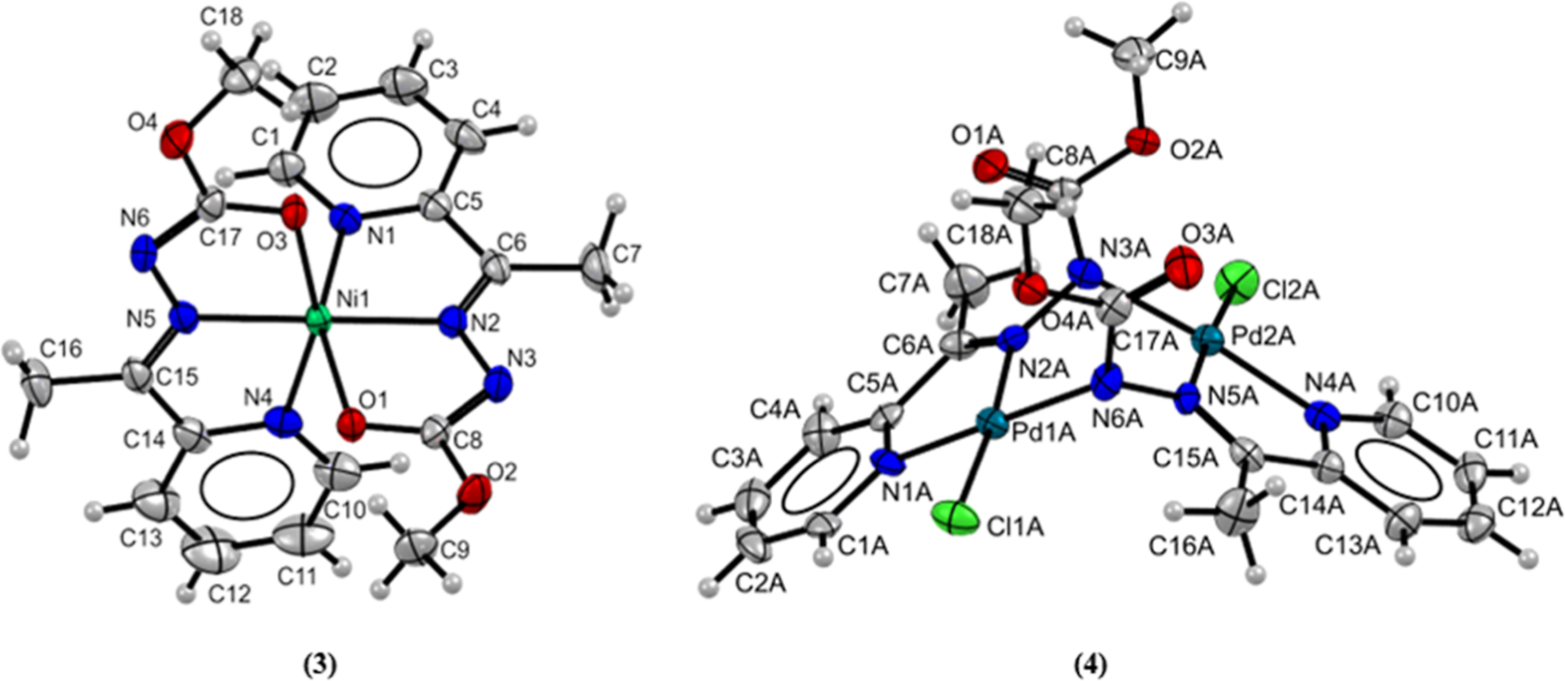
Molecular structure of **(3)** and **(4)** with crystallographic labeling. Thermal ellipsoids with 30% of probability.

Complex **(4)** is binuclear and presents an unusual coordination mode for the carbazate ligand (Figure 4). The Pd1 atom exhibits a square-planar geometry and is coordinated to a chloride ion, two nitrogen atoms of one ligand molecule, and one nitrogen atom of a second ligand molecule. The Pd2 atom is coordinated similarly to that of the Pd1 atom. The carbazate molecules coordinate with the palladium atom in its ketonic and deprotonated form, resulting in a binuclear structure with the formation of six-membered rings. The asymmetric unit of this compound consists of three molecules of the complex (A, B and C), and the carbon–oxygen bond lengths in the ketonic part of the carbazate range from 1.187(9) to 1.232(2) Å. In addition, the bond distances between nitrogen and carbon atoms of the ester range from 1.369(2) to 1.330(2) Å, indicating the presence of the ketonic form. These values closely resemble those of the free ligand and similar complexes.^32,33^

In general, tridentate ligands containing the pyridine moiety usually give rise to strained square-planar palladium­(II) complexes with the N­(py)–Pt–N­(cis) angles in the range 79.10–80.00° and N­(py)–Pt–N­(trans) angles in the range 172.20–174.40°. To verify quantitatively the geometry of Pd­(II) atoms in the complex **(4)**, the Okuniewski parameter (τ_4_) was used by the expression: $\tau_{4}=\frac{360^{{}^{\circ}}-\left(\beta +\alpha \right)}{360^{{}^{\circ}}-2\theta }$ , where α and β are the two largest angles of the coordination polyhedron and θ = 109.5°.^34^ Values of τ_4_ close to zero indicate square geometries and values close to one indicate tetrahedral geometries. All palladium atoms present in the asymmetric unit of complex **(4)** exhibited values between 0.073 and 0.102, which suggests the presence of square planar geometry as observed by bond angles close to 180°. Selected bond lengths and angles of **Hampc** and the complexes **(1–4)** are listed in Table 1.

**Table 1:** Selected Bond Lengths (Å) and Angles (°) for **Hapmc** and the Complexes **(1–4)** ^a^

bond lengths (Å)							
		(1)	(2)	(3)	(4)		
Hapmc		M=Cu and X=Cl	M=Cu and X=Br	M=Ni	M=Pd and X=Cl (A) (B) (C)		
M–X1		2.233(2)	2.358(8)		2.292(2)	2.296(3)	2.298(2)
M–X1’		2.743(3)					
M–X2			2.578(9)		2.298(2)	2.304(3)	2.283(2)
M–N1		2.010(4)	2.033(4)	2.065(2)	2.006(7)	1.992(7)	2.009(7)
M–N2		1.928(4)	1.972(4)	1.985(2)	1.990(6)	2.013(7)	1.998(7)
M–N3					2.017(7)	1.999(7)	1.996(7)
M–N4				2.086(2)	2.002(7)	2.010(7)	2.012(7)
M–N5				1.986(2)	2.003(6)	1.992(7)	2.006(7)
M–N6					2.009(7)	1.975(7)	1.988(7)
M–O1		1.993(4)	2.106(3)	2.116(2)			
C8–O1	1.213(2)	1.265(6)	1.232(6)	1.247(3)	1.187(9)	1.212(2)	1.216(2)
N2–N3	1.377(2)	1.379(6)	1.359(5)	1.378(3)	1.411(9)	1.367(9)	1.414(9)
N3–C8	1.348(2)	1.338(6)	1.353(6)	1.333(3)	1.369(2)	1.364(2)	1.343(2)
bond angles (°)							
X1–M–X2			106.97(3)				
X1–M–X1’		93.10(2)					
X1–M–O1		98.30(2)	97.59(9)				
N1–M–O1		159.03(2)	153.92(2)	155.45(8)			
N4–M–O3				155.61(9)			
N4–M–O3				177.21(2)			
N2–M–X1		169.85(2)	155.85(2)		175.00(2)	174.90(2)	175.50(2)
N2–M–N6					93.70(3)	94.50(3)	93.50(3)
N1–M–X1		100.00(2)	99.10(2)		96.40(2)	95.30(2)	97.70(2)
N2–M–N1		81.03(2)	78.83(2)	78.78(9)	79.80(3)	80.00(3)	79.30(3)
N2–M–O1		79.17(2)	77.73(2)	76.69(8)			
O1–M–O3				94.78(7)			
O3–M–N5				76.95(8)			
O1–C8–O2	123.67(2)	119.00(4)	126.10(4)	119.80(3)	125.30(8)	123.30(2)	123.90(9)
N3–C8–O2	113.92(2)	112.50(5)	111.40(4)	111.10(2)	107.30(8)	110.00(8)	108.40(8)

a Symmetry operations: ’–*x* + 3/2, −*y* + 3/2, −*z* + 1.

X-ray data of the complex **(4)** revealed the formation of a one-dimensional chain along the crystallographic axis *c*, which is formed by different π···π stacking interactions between the pyridinic rings of the ligand molecules. The lengths of these intermolecular interactions range from 3.517 to 3.699 Å. Figure 5 shows the unidimensional chain formed in the crystal lattice of complex **(4)**.

**Figure 5:**
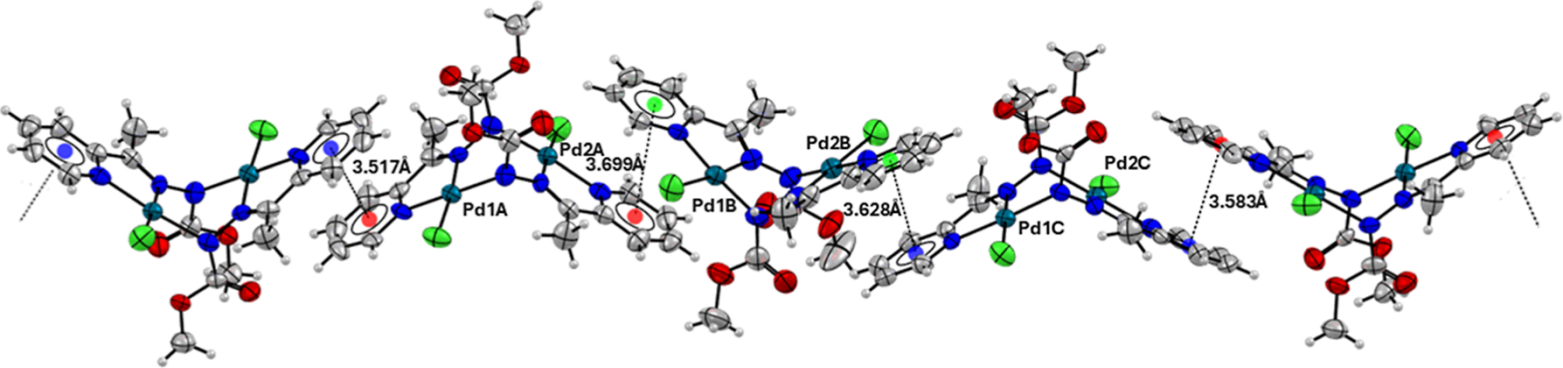
Unidimensional chain along the crystallographic axis *c* formed by π···π stacking interactions in complex **(4)**.

### 2.2 Hirshfeld Surface

The Hirshfeld Surface analysis (HS) was used to evaluate the intermolecular interactions present in the crystal lattice. The HS has various functions that enable the examination of different properties and the quantitative and qualitative study of the interactions in the crystal.^35^ The *d*_norm_ function was used to qualitatively evaluate the intermolecular contacts around the compound by a color pattern. The red color represents contacts smaller than the sum of the van der Waals radius of the atoms involved, the blue color represents contacts bigger than the sum of the van der Waals radius, and the white color represents contacts close to the sum of the van der Waals radius. The *d*_norm_ function of HS for the synthesized compounds is presented in Figure 6.

**Figure 6:**
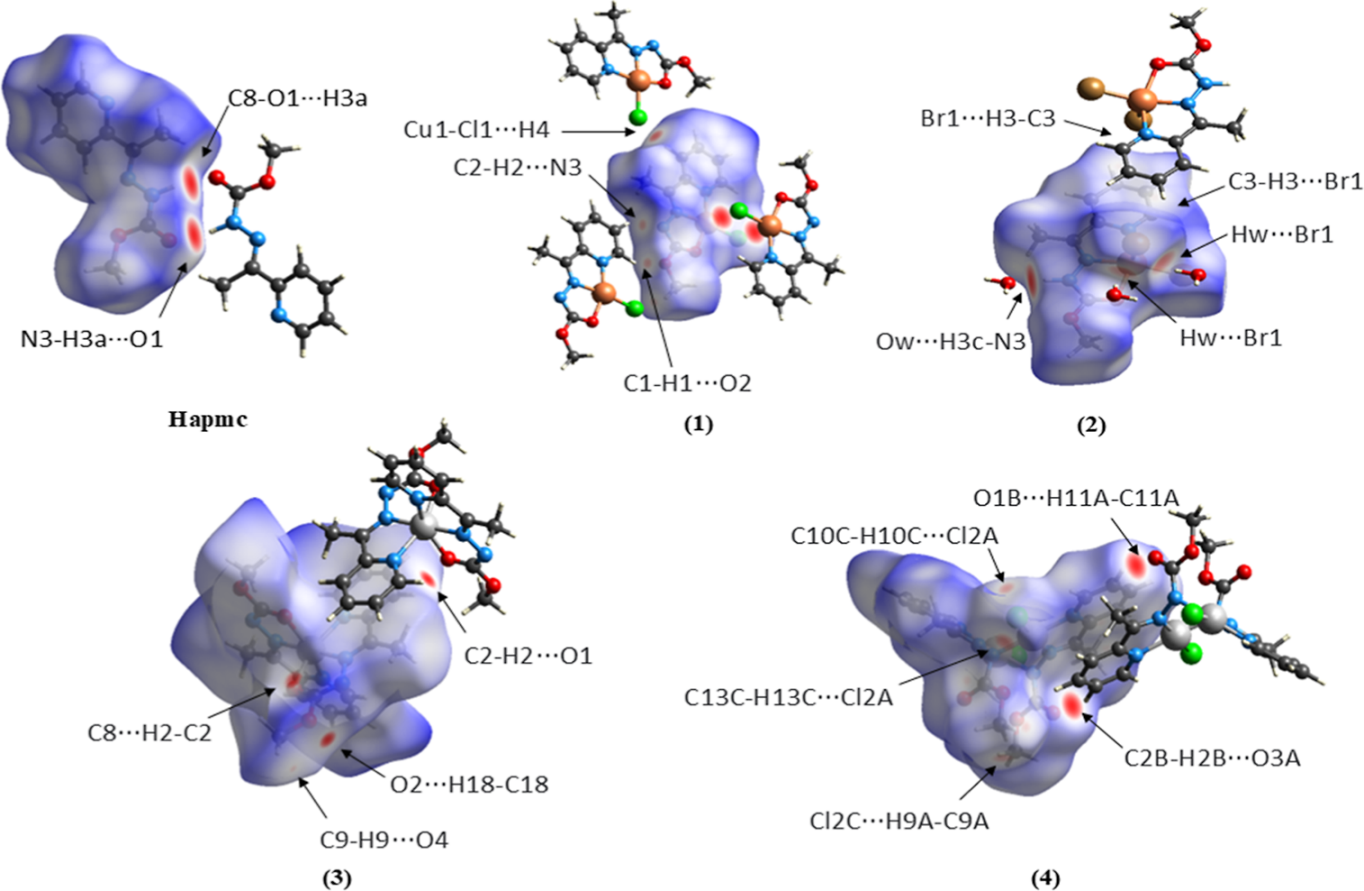
Hirshfeld surface of **Hapmc** and the complexes **(1–4)** mapped with *d*_norm_ function.

It is possible to observe in **Hapmc** strong interactions between N3–H3a···O1 with a distance of 2.07 Å. In the complexes **(1)**, **(2)**, and **(4)** are observed red contacts referring to the weak hydrogen bonds C–H···N, C–H···O e C–H···Cl/Br. Additionally, in complex **(2)**, the solvent water molecules present in the unit cell exhibit different contacts smaller than the sum of the van der Waals radius between N3–H3a···O3 and Cu1–Br2···H3b with distances of 1.75 and 2.32 Å, respectively. Complex **(3)** exhibited only weak hydrogen bonds contacts C···H–C and O···H–C. In the case of complex **(4)**, the molecule containing the atoms Pd1 and Pd2 was used as a reference, although the other two molecules in the asymmetric unit showed similar contacts, and their surfaces are represented in Figure 7.

**Figure 7:**
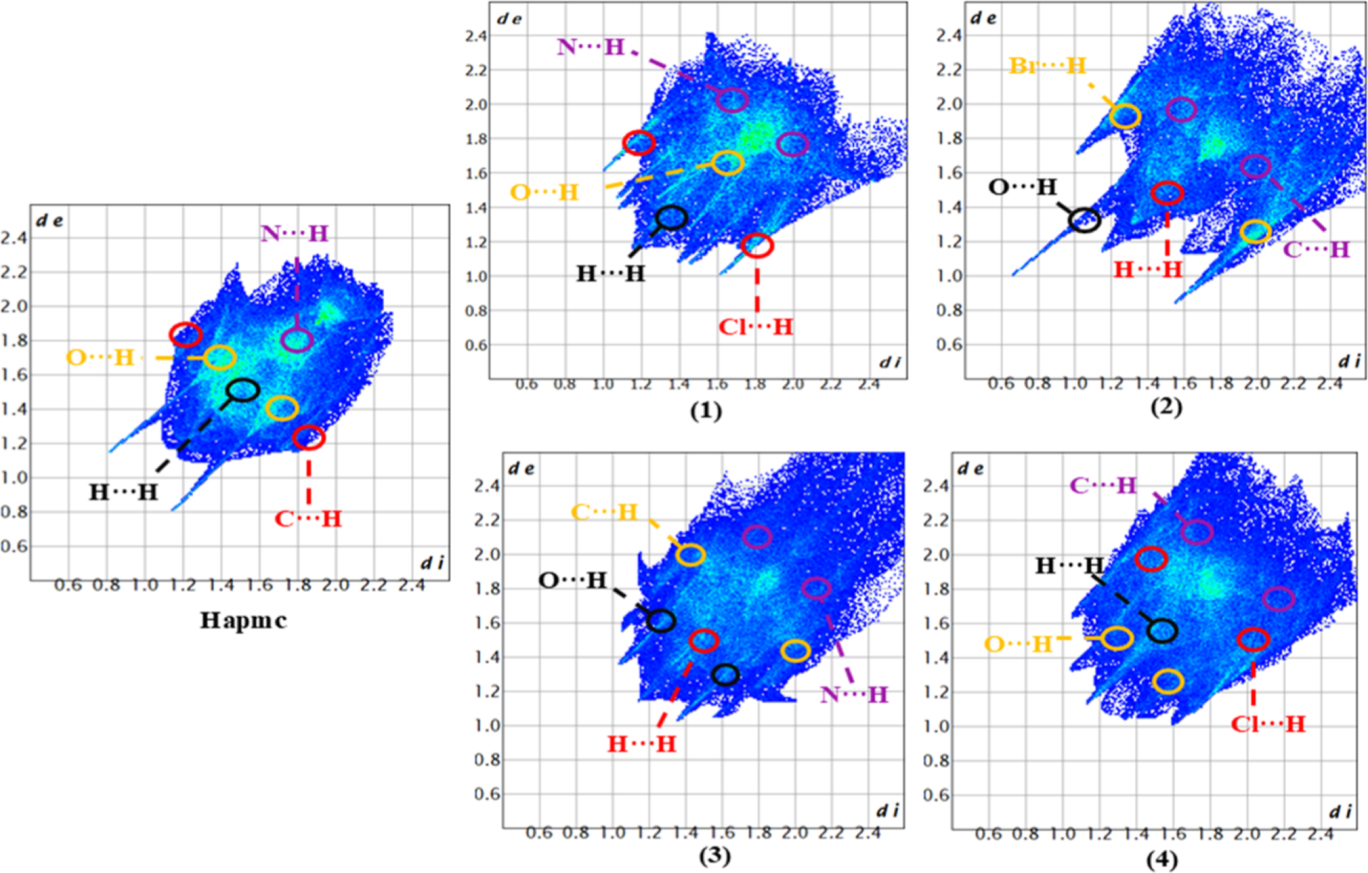
Fingerprint plot of **Hapmc** and complexes **(1–4)**, highlighting the area with the highest concentration of dots in each type of close contact.

The fingerprint plots show the intensity of intermolecular interactions by the colored area and by a color pattern. The color varies from blue to red, indicating few to many dots, respectively. In synthesized compounds, the contact that most contributed to the formation of the crystal lattice was the H···H contacts range of 53.3–31.4%, except in complex **(2)** with 34.2%, which has the H···Br interactions as the one that contributed most, probably due to the interaction between the bromide ions and the water molecules present in the unit cell. Figure 7 shows the fingerprint plot of **Hapmc** and complexes **(1–4)**, highlighting the area with the highest concentration of dots in each contact. Figure 8 presents a graph with the percentage contribution of each interaction to the formation of the crystal lattice for each compound (Figures S1–S7).

**Figure 8:**
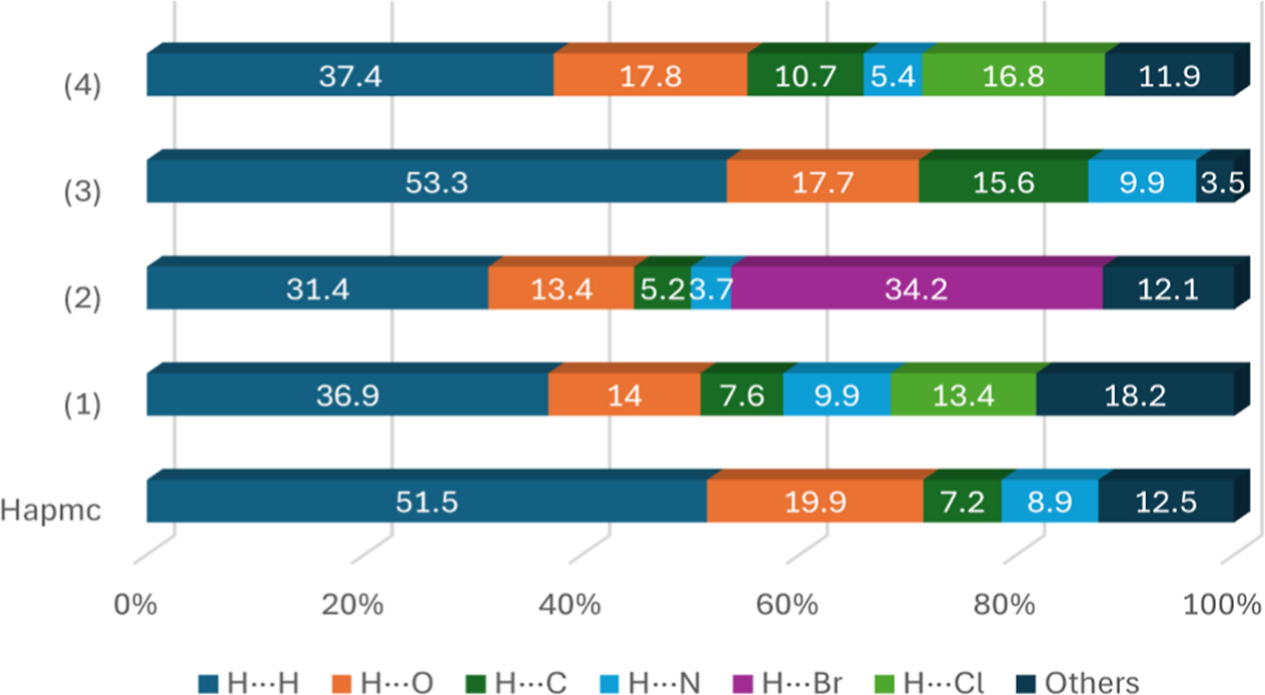
Percentage contribution of each interaction to the crystal lattice formation for **Hapmc** and complexes **(1–4)**.

The shape index function is useful to evaluate the presence of π···π stacking interactions. These interactions are indicated by contact between the vertices of a pair of red and blue triangles on the mapped surface. The shape index function revealed that only the complexes **(1**, **2**, and **4)** show π···π stacking interactions (Figure 9). In complexes **(1)** and **(4)**, the π···π stacking occurs over the pyridinic rings with a distance of 3.776 Å for complex **(1)** and between 3.514 and 3.669 Å in complex **(4)**. These interactions in complex **(4)** are responsible for the formation of the unidimensional chain (Figure 3). Complex **(2)** exhibits two π···π stacking on its surface, resulting from the interactions between the π orbitals of the pyridinic ring with the π orbitals of the five-membered ring formed by the coordination of the ligand molecule with the metal center with a distance of 3.460 Å.

**Figure 9:**
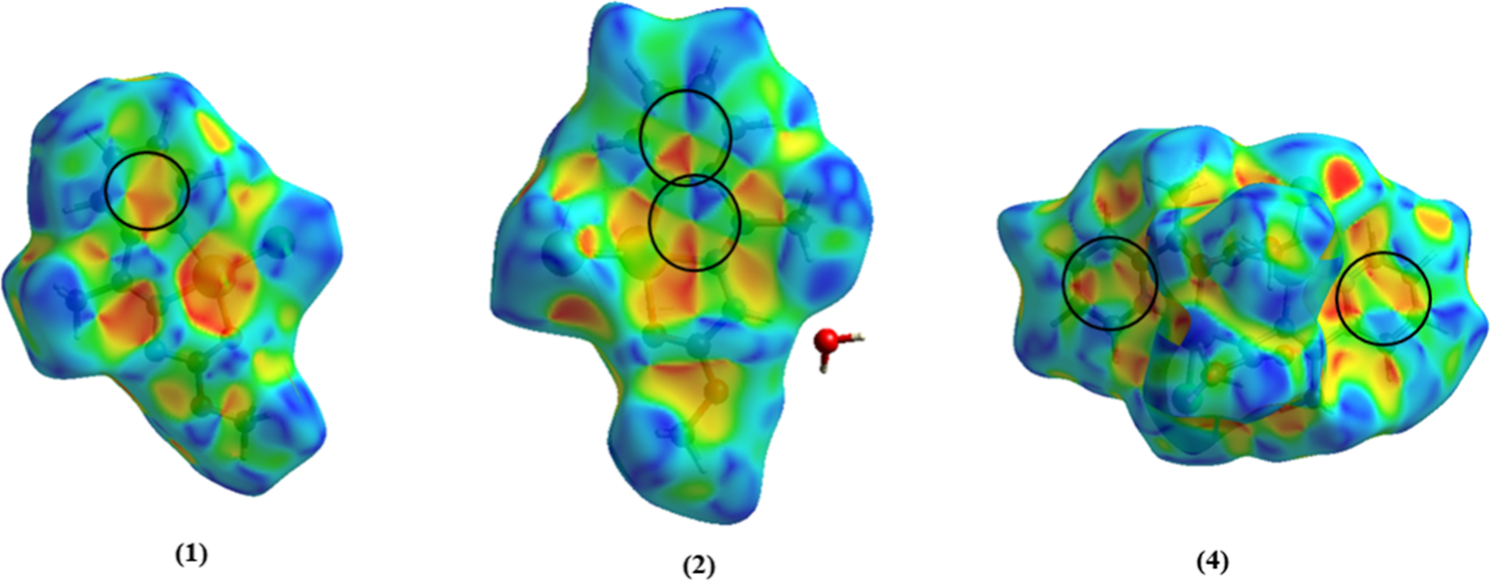
Hirshfeld surface in shape index function for the complexes **(1)**, **(2)** and **(4)** showing the π···π stacking interactions.

### 2.3 Spectroscopy Analysis

The IR spectra of **Hapmc** and complexes **(1–4)** were obtained using the KBr pallets technique (Figures S8–S12). Table 2 presents the main bands identified in the spectra of the synthesized compounds.

**Table 2:** Main Bands in IR Spectra of Complexes **(1-4)** and Ligand **Hapmc**

	Hapmc	(1)	(2)	(3)	(4)
ν C=O	1705		1654		1662
ν C–O	1302	1305	1316	1342	1305
ν N–N	1056	1038	1040	1029	1046
ν C=N		1599	1598	1594	1602
ν C=N_py_	1577	1562	1562	1558	1574
ν N–H	3246		3377		

The spectrum of **Hapmc** exhibits all expected bands corresponding to the organic functions present in the molecule, except the stretch C6=N2 bond. This signal may be overlapping with the signal of the ν­(C=O) due to a large band present in 1705 cm^–1^. In the region of 3246 cm^–1^, a band of (N–H) is observed, while the stretching vibration of N–H is observed at 1755 cm^–1^. The IR data corroborate with the data obtained through X-ray diffraction and are under similar works in the literature.^14,36^ The complexes **(1)** and **(3)** spectra exhibit similar behavior and the ν­(C=O) and ν­(N–H) stretching was not observed. This indicates that in both complexes, the ligand coordinates to the metal center in the enolic and deprotonated form. Other evidence of complexation is the band shift of atoms that are coordinated to the metal center. Specifically, a shift from 1577 cm^–1^ to 1562 cm^–1^ was noted for the pyridinic band, while the azomethine band showed a shift from 1705 cm^–1^ to 1599 cm^–1^. For complexes **(2)** and **(4)**, the **Hapmc** ligand is coordinated in ketonic form as evidenced by the presence of ν­(C=O) at 1654 cm^–1^ and 1662 cm^–1^ for complexes **(2)** and **(4)**, respectively. Also is observed a band in complex **(2)** at 3377 cm^–1^ that is associated with ν­(N–H), this indicates that the ligand is protonated. However, it is not observed in the complex **(4)** spectrum because the ligand is coordinated by the three nitrogen atoms of the deprotonated ligand molecules. These results follow similar work reported in the literature.^25,37–39^

The electronic spectra of the synthesized compounds were obtained in DMF and MeOH, except for complex **(4)** due to insufficient solubility in MeOH (Figures S13 and S14), and Table S1 shows the absorption values of the bands observed in the electronic spectra of **Hapmc** and complexes **(1–4)**. In the **Hapmc** spectrum, bands at 283 and 286 nm were observed in MeOH and DMF, respectively. This band corresponds to π→π* transition from the azomethine and the pyridinic ring. In the spectra of the complexes, a bathochromic shift is noted for the π→π* transitions, occurring at 283 and 285 nm in MeOH and 287 and 293 nm in DMF. The bands between 301 and 315 nm in MeOH and 342 and 368 nm in DMF can be assigned to charge transfer in the coordination sites of the ligand with the metal center. Complex (4) showed a band in 400 nm that also can be assigned to charge transfer ligand–metal.^25,40^ A new band was observed at a higher concentration (2 × 10^–3^ mol/L). This band appears within the wavelength range of 740 and 816 nm in MeOH and 749 and 810 nm in DMF. It is attributed to d–d transitions in complexes **(1–3)**. The d-d transition band for **(4)** is due to the d^8^ electronic configuration and one pair of electrons from the ligand molecule occupying the last d orbital of the metal and the prohibited transition by Laporte.

ESI­(+)-MS and ESI­(+)-MS/MS analyses were performed on all synthesized compounds. Complexes **(1)**, **(2)**, and **(4)** showed the formation of molecular ions after the loss of one halogen ion, yielding *m*/*z* of 255.0062, 334.9328, and 630.9309, respectively. In the case of **Hapmc** and complex **(3)**, the molecular ions were formed as [M + H]^+^, resulting in *m*/*z* of 194.0924 and 443.0965, respectively. The ESI­(+)-MS/MS spectra of the synthesized compound have displayed an expected and similar fragmentation pattern (Figures S15–S24).

The NMR analyses were conducted to confirm the synthesis of the **Hapmc** and to evaluate which tautomer is exhibited in the solution. The ^1^H and ^13^C NMR spectra were obtained in DMSO-*d*_6_ and are represented in Figures S25 and S26. In the ^1^H spectra, the singlet at 2.30 ppm corresponds to the CH_3_ group attached to azomethine. The singlet at 3.74 ppm is attributed to the hydrogen atoms of CH_3_ of the ester portion. The signals of the aromatic hydrogens are between 7.38 and 8.57 ppm and present an integral equal to 4. The signal at 10.34 ppm is characteristic of the hydrogen atom in the NH group. The absence of a signal associated with the OH group indicates that the ligand is in the ketonic form in solution; this behavior is observed in similar works in the literature.^25,39^ In the ^13^C spectra, nine signals were observed. The CH_3_ signals at 11.92 and 51.99 ppm are attributed to the methyl attached to the azomethine and ester, respectively. Between 119.88 and 148.38 ppm are the signals of the carbons of the pyridine ring. The carbon bond to the azomethine group exhibits a signal at 154.37 ppm. The azomethine carbon shows a signal at 149.37 ppm, and 155.10 ppm is the signal of ester carbon.

The ^1^H NMR spectrum of complex **(4)** (Figure S27) was obtained in DMSO-*d*_6_. A significant observation was the disappearance of the signal around 10.34 ppm, which is associated with the hydrogen atom of the NH group, indicating the deprotonation of the ligand and the coordination with the metal ion. This finding was supported by the X-ray diffraction and mass spectrometry data. The presence of the metal caused a shift in the remaining signals. The signals associated with the CH_3_ group shifted from 2.30 and 3.74 ppm to 2.38 and 2.54 ppm, respectively. A shift was also observed in the signals related to the hydrogen atoms of the aromatic ring, shifting from the region of 7.38–8.57 to 7.87–8.98 ppm. The integrals of the spectrum of complex **(4)** revealed the presence of 12 hydrogen atoms, suggesting that the complex is in a symmetric dimer form in solution; this same behavior was also confirmed by mass spectrometry analysis.

### 2.4 In Vitro Cytotoxicity

DMSO solutions (10^–3^ M) of the **Hapmc** and metal­(II) complexes were subjected to molar conductance measurements at room temperature over 48 h, and the observed values are presented in Table S2. The carbazate ligand and complexes **(3)** and **(4)** showed low molar conductance values, which indicates that they are nonelectrolytes and further confirms the absence of external counterions. In contrast, complexes **(1)** and **(2)** showed evidence of labilization of the chloride and bromide ligands in DMSO, leading to the formation of cationic species, and this behavior corroborates the results observed in the mass spectrometry analysis.^41^

The biological activities of **Hapmc**, the metal complexes **(1–4)**, and Cisplatin (reference drug) were evaluated in vitro against human cancer cells of A549 lung cancer, MCF-7 breast cancer, A2780cis cisplatin-resistant ovarian cancer, and noncancerous human lung cell MRC-5. The compounds’ proliferative ability in 48 h was significantly reduced with increasing concentration (Table 3 and Figure 10). The results revealed that metal complexes have better cytotoxicity activity than the free ligand, showing the importance of complexation reactions with metal ions.

**Table 3:** Cytotoxic Activity of **Hapmc**, Metal Complexes **(1–4)**, and Cisplatin^a^

	IC_50_ (μM)			
compound	MCF-7	A549	A2780cis	MRC-5
**Hapmc**	>100	>100	>100	>100
**(1)**	>100	(70.8 ± 0.2)	(14.74 ± 2.13)	(21.49 ± 0.24)
**(2)**	(48.03 ± 4.12)	(49.05 ± 0.35)	(20.35 ± 4.06)	>100
**(3)**	(50.66 ± 1.35)	(3.09 ± 0.07)	>100	>100
**(4)**	>25	>25	>25	>25
**Cisplatin**	(13.98 ± 2.02)	(11.54 ± 1.19)	(37.03 ± 5.11)	(29.09 ± 0.78)

a Results are presented as IC_50_ values (μM) ± SD in 48 h (values estimated by nonlinear regression of data from viability assessment).

**Figure 10:**
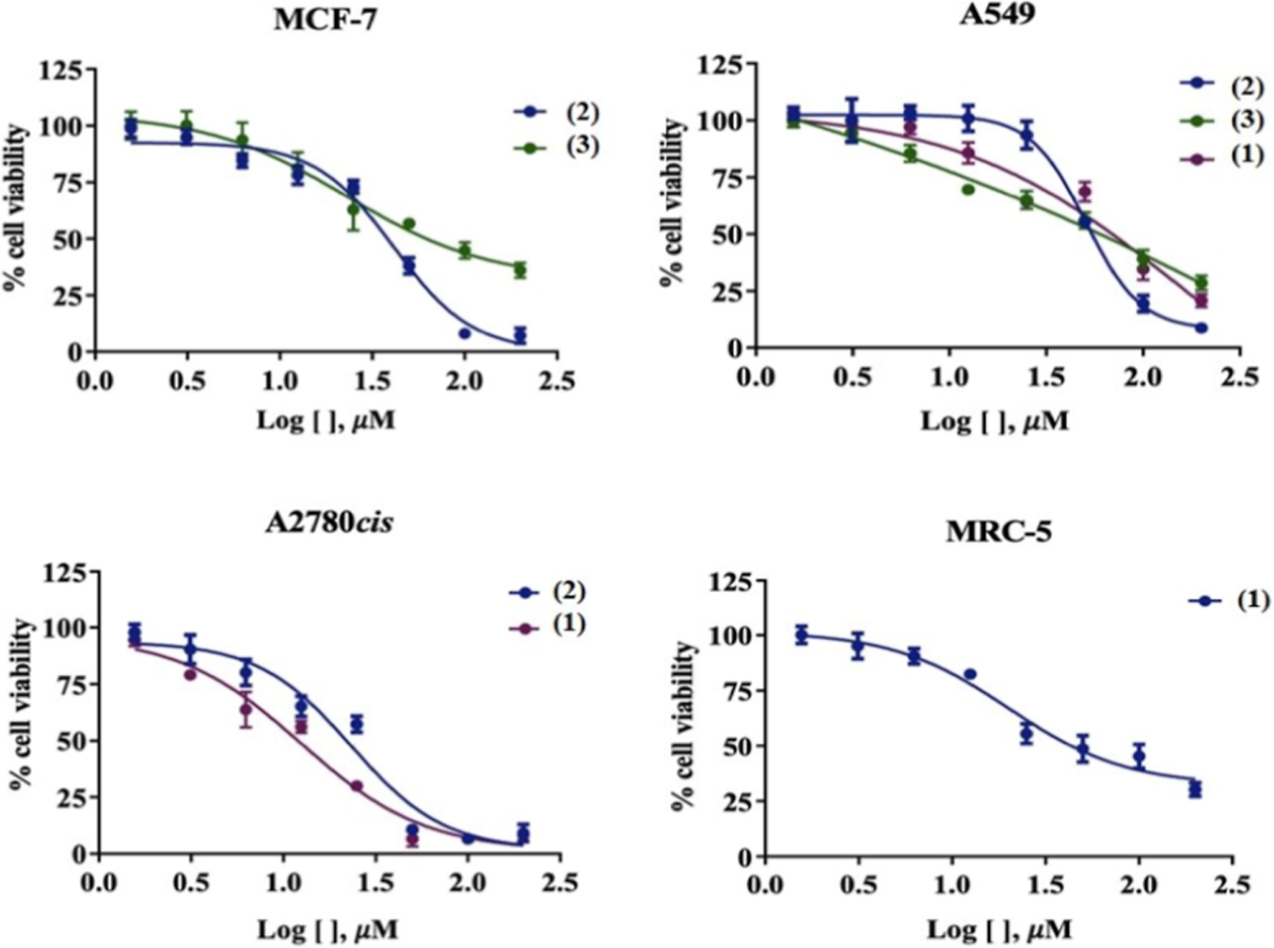
Evaluation of cytotoxic effects by MTT assay in 48 h. DMSO at 0.5% did not affect the cell viability of the cell lines.

The biological assays demonstrated that ligand complexation enhances its cytotoxicity activity while maintaining selectivity. The free ligand exhibited an IC_50_ above 100 μM for all tested cell lines, indicating low cytotoxicity. In contrast, the metal complexes showed increased efficacy. Complex **(1)** was active against all tested cancer cell lines except MCF-7, with significant effectiveness against A2780cis, where it outperformed the reference drug. Complex **(2)** exhibited broad activity against cancer cells without affecting healthy MRC-5 cells, demonstrating greater selectivity. Additionally, complex **(2)** was more cytotoxic than cisplatin against A2780cis, a particularly resistant cell line, reinforcing the need for alternative therapies with improved efficacy and selectivity. Among the studied complexes, complex **(3)** exhibited the most promising results, with an IC_50_ of 3.09 μM against A549 lung cancer cells, while maintaining low toxicity toward healthy cells. Despite solubility limitations, complex **(4)** demonstrated higher activity than the free ligand, suggesting potential for further optimization. These findings highlight the impact of structural modifications, such as halide substitution and ligand protonation, on the biological activity and selectivity of metal complexes. The results align with previous literature reports, reinforcing the relevance of these compounds in the search for new anticancer agents with improved therapeutic profiles.^3,21,42^

## 3 Materials and Methods

The solvents and reagents were purchased from commercial sources and used without purification. The FT-IR spectra of the compounds were obtained from KBr pellets, using an FT-IR Varian 640 spectrometer in the range of 4000–400 cm^–1^. Electrospray ionization mass spectrometry analysis (ESI-MS) was performed on an AB Sciex Triple TOF 5600+ mass spectrometer in positive mode, with a voltage of 5500 V and source temperature of 200 °C. ^1^H nuclear magnetic resonance spectra were collected on a Varian Mercury Plus (600 MHz), with TMS as internal reference and DMSO-*d*_6_ as solvent. Elemental analysis of CHN was performed with a PerkinElmer/Series II 2400 analyzer. Electronic spectra were obtained using a UV–vis–NIR Varian Cary 5000 spectrophotometer using 20 μM or 1 mM solutions prepared in methanol or DMF. The molar conductance of a 10^–3^ M solution of ligand and metal complexes in DMSO was measured at room temperature using a Quimis Q405 M conduct meter.

### 3.1 Synthesis of 2-Acetylpyridine-methylcarbazate Ligand (Hapmc)

The ligand was synthesized using methylcarbazate (2 mmol, 180.16 mg) and 2-acetylpyridine (2 mmol, 0.2 mL) with 10 mL of ethanol. The mixture was refluxed and heated for 3 h. The colorless solution was slowly evaporated, and the white product was filtered and recrystallized in MeOH. After slow evaporation at room temperature, colorless crystals suitable for single-crystal X-ray diffraction were obtained. Yield: 94% (362.98 mg). mp: 127–131 °C. Selected IR bands (KBr, ν/cm^–1^): ν­(N–H) 3246, ν­(C=O) 1705, ν­(C=N) 1705, ν­(C=N_py_) 1577, ν­(N–N) 1056. NMR ^1^H (DMSO-*d*_6_, δ, ppm): 2.30 (s, 3H, CH_3_); 3.74 (s, 3H, CH_3_); 7.38 (dd, *J* = 7.4; 4.8; 1.2 Hz, 1H, CH_Ar_); 7.82 (td, *J* = 8.1; 7.4; 1.8 Hz, 1H, CH_Ar_); 7.99 (d, *J* = 8.1; 1.1; 1.1 Hz, 1H, CH_Ar_); 8.57 (dq, *J* = 4.9; 1.8; 1.0 Hz, 1H, CH_Ar_); 10.34 (s, 1H, NH). RMN ^13^C (DMSO-*d*_6_, δ, ppm): 11.92 (C^7^); 51.99 (C^9^); 119.88 (C^4^); 123.61 (C^2^); 136.40 (C^3^); 148.38 (C^1^); 149.37 (C^6^); 154.43 (C^5^); 155.10 (C^8^). UV–vis (MeOH): λ_max_ = 283 nm. UV–vis (DMF): λ_max_ = 286 nm. Elemental Anal. Calcd for C_9_H_11_N_3_O_2_ (%): C 55.95; H 5.74; N 21.75 and. Found: C, 55.53; H 5.57; N 21.64. ESI-MS [M + H]^+^ (calcd, found, *m*/*z*) = 194.0924/194.0923.

### 3.2 Synthesis of Synthesis of [Cu­(apmc)­Cl]_2_ **(1)**

A solution with 0.1 mmol of CuCl_2_ (13.5 mg) in 5 mL of MeOH was added to a Hapmc 0.1 mmol (19.3 mg) solution in 5 mL MeOH. The mixture was refluxed for 2 h. Green crystals suitable for X-ray diffraction were obtained from the mother solution after slow evaporation at room temperature. Yield: 39% (11.32 mg). mp: 164–166 °C. Selected IR bands (KBr, ν/cm-1): ν­(C=N) 1602, ν­(C=Npy) 1520, ν­(C–O) 1293, ν­(N–N) 1038. UV–vis (MeOH): λ_max_ = 240 nm; (DMF): λ_max_ = 287 nm. Elemental Anal. Calcd for C18H20O4N6Cl2Cu2 (%): C 37.12; H 3.46; N 14.43 and. Found (%): C 37.58; H 3.64; N 14.09. ESI-MS [M + H]+ (calcd, found, *m*/*z*) = 250.0063/250.0065.

### 3.3 Synthesis of [Cu­(Hapmc)­Br_2_] **(2)**

A solution with 0.1 mmol of CuBr_2_ (22.3 mg) in 5 mL of MeOH was added to a Hapmc 0.1 mmol (19.3 mg) solution in 5 mL of MeOH. The mixture was refluxed for 2 h. The final solution evaporated under refrigeration slowly and after a few months, green crystals suitable for X-ray diffraction were obtained. Yield: 42% (18.10 mg). mp: 202–206 °C. Selected IR bands (KBr, ν/cm^–1^): ν­(C=N) 1598, ν­(C=N_py_) 1562, ν­(C=O) 1654, ν­(N–N) 1040, ν­(N–H) 3377. UV–vis (MeOH): λ_max_ = 241 nm; (DMF): λ_max_ = 289 nm. Elemental Anal. Calcd for C_9_H_11_O_2_N_3_Br_2_Cu (%): C 25.94; H 2.66; N 10.09 and. Found (%): C 25.75; H. 2.56; N. 9,61. ESI-MS [M + H]^+^ (calcd, found, *m*/*z*) = 336.9305/336.9308.

### 3.4 Synthesis of [Ni­(apmc)_2_] **(3)**

A solution with 0.2 mmol of Hapmc in 5 mL of MeOH, with two drops of triethylamine, was added to a solution containing 0.1 mmol (23.70 mg) of NiCl_2_·6H_2_O in 5 mL of MeOH. The mixture was refluxed for 2 h. Yellow crystals suitable for X-ray diffraction were obtained from the mother solution after slow evaporation at room temperature. Yield: 43% (19.10 mg). Selected IR bands (KBr, ν/cm^–1^): ν­(C=N) 1598, ν­(C=N_py_) 1558, ν­(N–N) 1029. UV–vis (MeOH): λ_max_ = 350 nm; (DMF): λ_max_ = 368 nm. Elemental Anal. Calcd for C_18_H_20_O_4_N_6_Ni (%): C 48.74; H 4.55; N 18.97 and. Found (%): C 48.41; H 4.67; N 18.55. ESI-MS [M + H]^+^ (calcd, found, *m*/*z*) = 443.09723/443.0975.

### 3.5 Synthesis of [Pd­(apmc)­Cl]_2_ **(4)**

0.1 mmol of Hapmc was solubilized in a mixture of 10 mL of MeOH and 5 mL of acetonitrile and was added to the solution of 0.1 mmol (17.70 mg) of PdCl_2_. The mixture was refluxed for 2 h. The yellow solid was filtered and recrystallized in DMF, and after a few days at room temperature, orange crystals suitable for X-ray diffraction were obtained. Yield: 39% (13.00 mg). Selected IR bands (KBr, ν/cm^–1^): ν­(C=O) 1662, ν­(C=N) 1602, ν­(C=N_py_) 1578, ν­(N–N) 1046. NMR ^1^H (DMSO-*d*_6_, δ, ppm): 2.38 (s, 6H, CH_3_); 3.54 (s, 6H, CH_3_); 7.87 (ddd, *J* = 7.5; 5.7; 1.4 Hz, 2H, CH_Ar_); 8.23 (d, *J* = 8.1 Hz, 2H, CH_Ar_); 8.38 (td, *J* = 7.5; 1.4 Hz, 2H, CH_Ar_); 8.98 (m, *J* = 9–8.95 Hz, 2H, CH_Ar_). UV–vis (DMF): 272 nm. Elemental Anal. Calcd for C_18_H_20_O_4_N_6_Cl_2_Pd_2_ (%): C 32.36; H 3.02; N 12.58 and. Found (%): C 32.41; H 2.99; N 12.48. ESI-MS [M + H]^+^ (calcd, found, *m*/*z*) = 630.93072/630.9300.

### 3.6 Crystal Structure Determination

X-ray data were gathered using a Bruker CCD SMART APEX II single crystal diffractometer equipped with Mo Kα radiation (0.71073 Å) at 296 K. SAINT^43^ processed the collected data, and absorption correction was performed using SADABS.^44^ The structures were solved through direct methods employing the SHELXS program,^45^ and the positions of non-hydrogen atoms were determined using subsequent Fourier-difference map analyses. Refinement was carried out using SHELXL-2018 with Least Squares minimization.^46^ Olex2 was utilized for both the solution and refinement of the structures, and Mercury was used for preparing the material for publication.^47,48^ The Solvent Mask function in Olex2 software was used for complex **(4)**. Crystal data, experimental details, and refinement results are summarized in Table S3.

### 3.7 Hirshfeld Surface

The calculations of Hirshfeld surfaces (HS) using the shape index and dnorm functions, as well as the 2D fingerprint plots of the crystal structures, were performed using CrystalExplorer 21.5.^49^ These analyses were conducted based on crystallographic information files (CIFs) obtained through single-crystal X-ray diffraction. The dnorm function allows the visualization of contacts that contribute to the formation of the crystal lattice using a color scale: red for contacts shorter than the van der Waals radius and blue for contacts longer than the van der Waals radius. The 2D fingerprint plots are generated by combining the di and de functions, providing a quantitative evaluation of the contacts responsible for the formation of the crystal lattice. The shape index surface is used to identify the presence of π···π stacking interactions, which are revealed by the contacts between the vertices of blue and red triangles.^35^

### 3.8 Anticancer Activity

#### 3.8.1 Cell Lines and Cell Culture

Human cell lines A549 (lung cancer, ATCC No. CCL-185), MRC-5 (nontumoral lung, ATCC No. CCL-171), MCF-7 (breast cancer, ATCC No. HTB-22), and A2780cis (cisplatin-resistant ovarian cancer, ECACC No. 93112517) were used to evaluate the cytotoxic activity of the metal complexes. The A549 and MRC-5 cell lines were cultured in DMEM (Dulbecco’s Modified Eagle’s Medium), while the MCF-7 and A2780cis cell lines were maintained in RPMI-1640 (Roswell Park Memorial Institute) medium. Both media were supplemented with 10% fetal bovine serum (FBS), penicillin (100 U/mL), streptomycin (100 mg/mL), and l-glutamine (2 mM). The cells were grown in monolayers using disposable 25 cm^2^ flasks and incubated at 37 °C in a 5% CO_2_ atmosphere.

#### 3.8.2 Cytotoxic Activity Analysis

The cytotoxicity of the compounds was determined using the MTT colorimetric assay (3-(4,5-dimethylthiazol-2-yl)-2,5-diphenyl tetrazolium bromide), as described by Mosmann.^50^ The cells were trypsinized for counting and concentration adjustment and then seeded in 96-well plates (1.5 × 10^4^ cells/well). The plates were incubated at 37 °C in a 5% CO_2_ atmosphere for 24 h to allow cell adhesion. Subsequently, the compounds were first dissolved in DMSO to prepare stock solutions, from which 0.75 μL aliquots were added to the wells of the plates to obtain different concentrations (200–1.56 μM), followed by incubation for 48 h. Cells treated with 0.5% DMSO were used as the control. After 48 h of treatment, 50 μL of MTT (0.6 mg/mL, Sigma-Aldrich) solubilized in PBS (Phosphate Buffered Saline) was added to each well and incubated for 4 h. The solutions in the wells were then removed, and formazan crystals were solubilized by adding 100 μL of DMSO per well. Absorbance was measured at 570 nm using a Varioskan LUX Multimode Microplate Reader (VL000D0). Experiments were performed in triplicate, and IC_50_ values (the concentration that inhibits 50% of cell growth) were determined from a dose–response curve using GraphPad Prism 8 software.^50^

## 4 Conclusions

One new carbazate **Hapmc** ligand and its four Cu­(II), Ni­(II), and Pd­(II) complexes were successfully synthesized and characterized by spectroscopic and physicochemical methods. Complexes **(1)** and **(4)** are binuclear structures, and the metal center is coordinated with the deprotonated ligand and chloride ions. Complex **(2)** was formed by coordinating the protonated ligand and two bromide ions. Complex **(3)** exhibited two deprotonated ligand molecules coordinated to the Ni­(II) atom. All complexes showed an *NNO* donor system, except complex **(4)** with an unusual coordination mode *NNN*. The biological study confirms that ligand complexation may enhance anticancer activity while maintaining selectivity. The free ligand (Hapmc) showed low anticancer activity, however, its complexes exhibited greater efficacy. Complex **(1)** was best performed against A2780cis, outperforming cisplatin, while complex **(2)** demonstrated a broad activity with good selectivity. Complex **(3)** showed the best result among all tested complexes, specifically against A549 lung cancer cells. Despite low solubility, complex **(4)** was more effective than the free ligand. This study showed the importance of metal complexation to increase the bioactivity of carbazate compounds. These findings emphasize the impact of structural modifications on biological activity and show the importance of the development of new metal-based anticancer agents.

## Supplementary Material













## Notes

Supplementary Tables, Figures, NMR, UV–vis, and IR spectra are as detailed in the text (PDF)
Crystallographic data (CIF)
Crystallographic data (CIF)
Crystallographic data (CIF)
Crystallographic data (CIF)
Crystallographic data (CIF)
