# Supplementary material for: Synthesis, Crystal Design and Anticancer Potential of Novel Cu(II), Ni(II), and Pd(II) Complexes with Carbazate Ligand
Source: ACS Omega. 2025 May 22;10(21):22125–36. doi: 10.1021/acsomega.5c02365 (PMC12138593; doi:10.1021/acsomega.5c02365)
Supplement: Supplementary file 1 [file ao5c02365_si_001.pdf]

Synthesis, crystal design and anticancer potential of novel Cu(II), Ni(II), and Pd(II) complexes with carbazate ligand

Supplementary Material

*Daniel J. de Siqueira[1], Mariana P. Viana[1], Katia M. Oliveira[1] and Claudia C. Gatto[1]**

[1]University of Brasilia, Institute of Chemistry, Laboratory of Inorganic Synthesis and Crystallography, Asa Norte, 70904-970, Brasília-Federal District, Brazil.

*Corresponding author: Claudia C. Gatto (e-mail address: ccgatto@gmail.com; Universidade de Brasilia, Institute of Chemistry, Laboratory of Inorganic Synthesis and Crystallography, Asa Norte, 70904-970, Brasília-Federal District, Brazil.

Summary:

Figure S1. Fingerprint plots for Hapmc. S4
Figure S2. Fingerprint plots for **(1)**. S4
Figure S3. Fingerprint plots for **(2)**. S5
Figure S4. Fingerprint plots for **(3)**. S5
Figure S5. Fingerprint plots for **(4)**, molecule containing Pd1A and Pd2A S6
Figure S6. Fingerprint plots for **(4)**, molecule containing Pd1B and Pd2B....................S6
Figure S7. Fingerprint plots for **(4)**, molecule containing Pd1C and Pd2C....................S7
Figure S8. IR spectra of ligand Hapmc..S7
Figure S9. IR spectra of complex **(1)**. S8
Figure S10. IR spectra of complex **(2)**. S8
Figure S11. IR spectra of complex **(3)**. S9
Figure S12. IR spectra of complex **(4)**. S9
Figure S13. UV-Vis spectra of complexes **(1-4)** and Hampc, in DMF and MeOH ($2x10^{-5}$ mol/L). S10
Figure S14. UV-Vis spectra of complexes **(1-4)** and Hampc, in DMF and MeOH ($2x10^{-3}$ mol/L). S10
Figure S15. ESI-MS(+) spectra of Hapmc..S11
Figure S16. ESI-MS(+) spectra of complex **(1)**. S11
Figure S17. ESI-MS(+) spectra of complex **(2)**. S12
Figure S18. ESI-MS(+) spectra of complex **(3)**. S12
Figure S19. ESI-MSMS(+) spectra of complex **(4)**. S13
Figure S20. ESI-MSMS(+) spectra of Hapmc. S13
Figure S21. ESI-MSMS(+) spectra of complex **(1)**. S14
Figure S22. ESI-MSMS(+) spectra of complex **(2)**. S14
Figure S23. ESI-MSMS(+) spectra of complex **(3)**. S15
Figure S24. ESI-MSMS(+) spectra of complex **(4)**. S15
Figure S25. ^{1}H NMR of ligand Hapmc..S16
Figure S26. ^{13}C NMR of ligand Hapmc. S17
Figure S27. ^{1}H NMR of complex **(4)**..S17
Figure S28. Hirshfeld surface of complex **(4)**. a) molecule containing Pd1B and Pd2B. b) molecule containing Pd1C and Pd2C...S18

Table S1. Absorption values of Hapmc and complexes (1-4) with molar absorptivity (logε) in parentheses...S18
Table S2. Molar conductivity data ($\Omega^{-1}.cm^{2}.mol^{-1}$) at 0, 24 and 48 hours of DMSO, **Hapmc** and complexes **(1–4)**……………….……………………………………………………...S18
Table S3. X-ray diffraction data collection and refinement parameters for **Hapmc** and the complexes **(1–4)**...S189

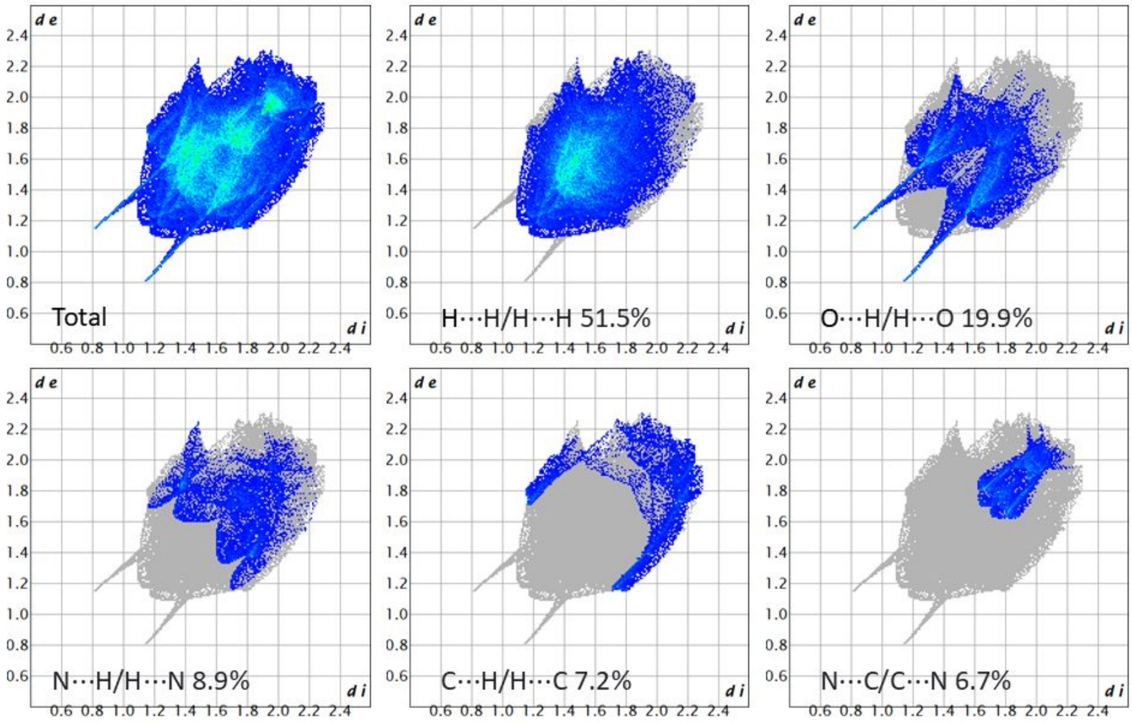

Figure S1. Fingerprint plots for **Hapmc**.

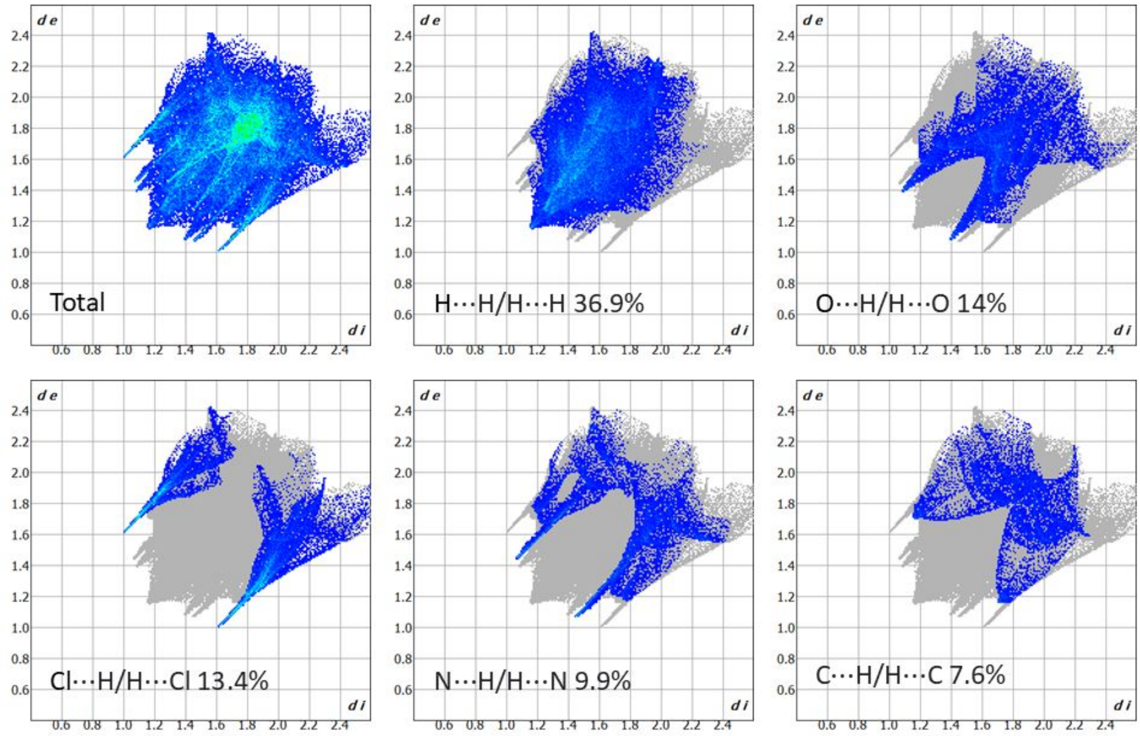

Figure S2. Fingerprint plots for **(1)**.

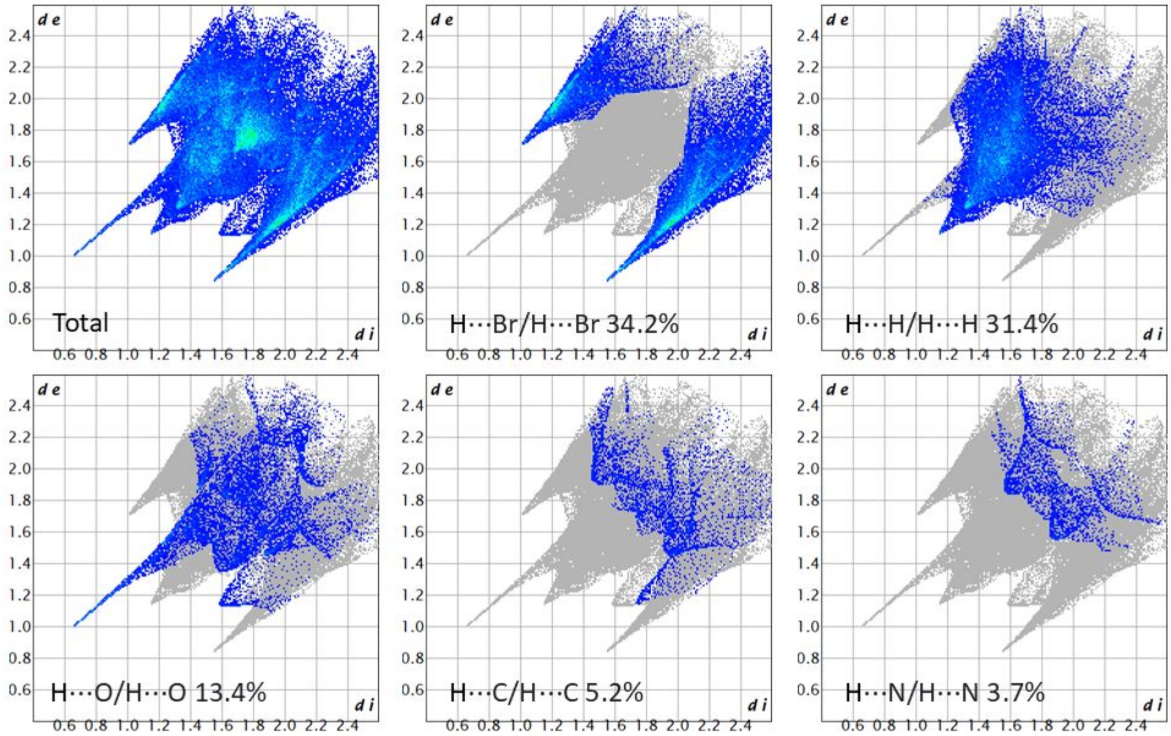

Figure S3. Fingerprint plots for **(2)**.

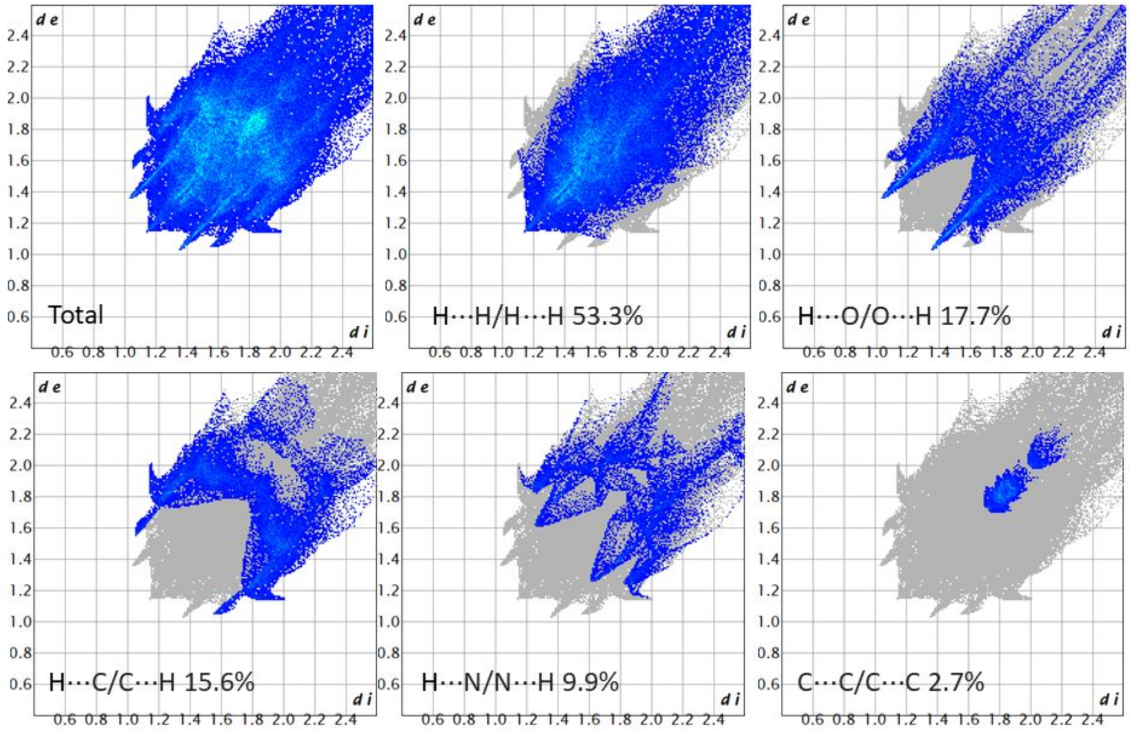

Figure S4. Fingerprint plots for **(3)**.

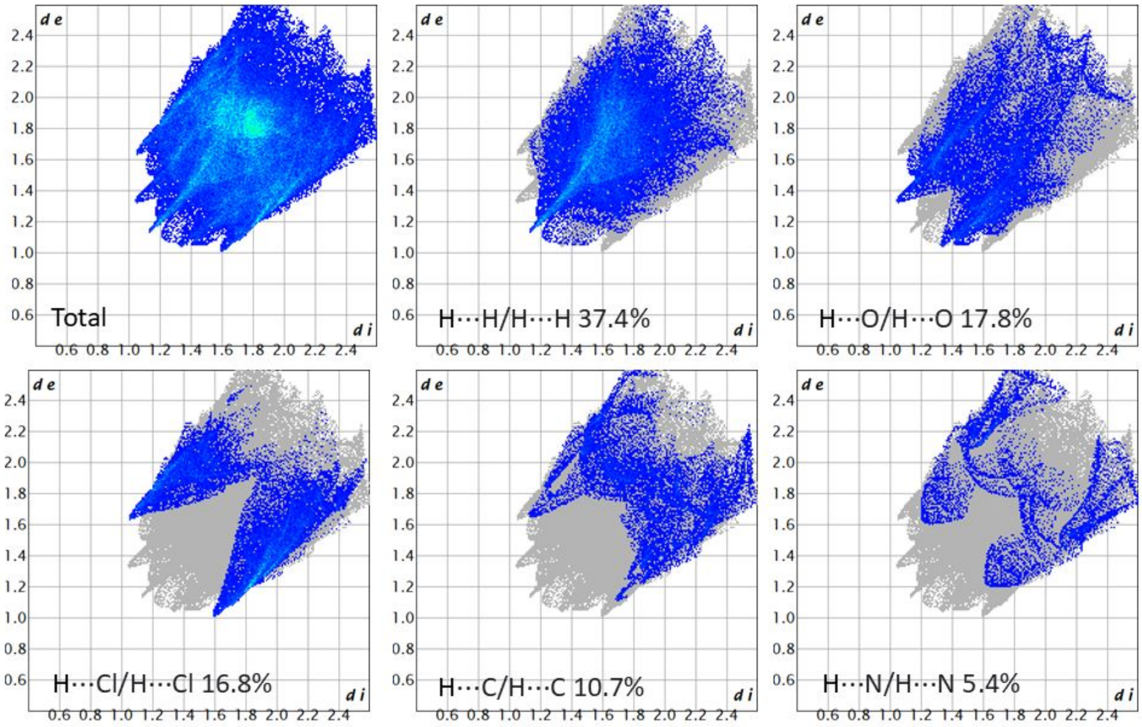

Figure S5. Fingerprint plots for **(4)**, molecule containing Pd1A and Pd2A.

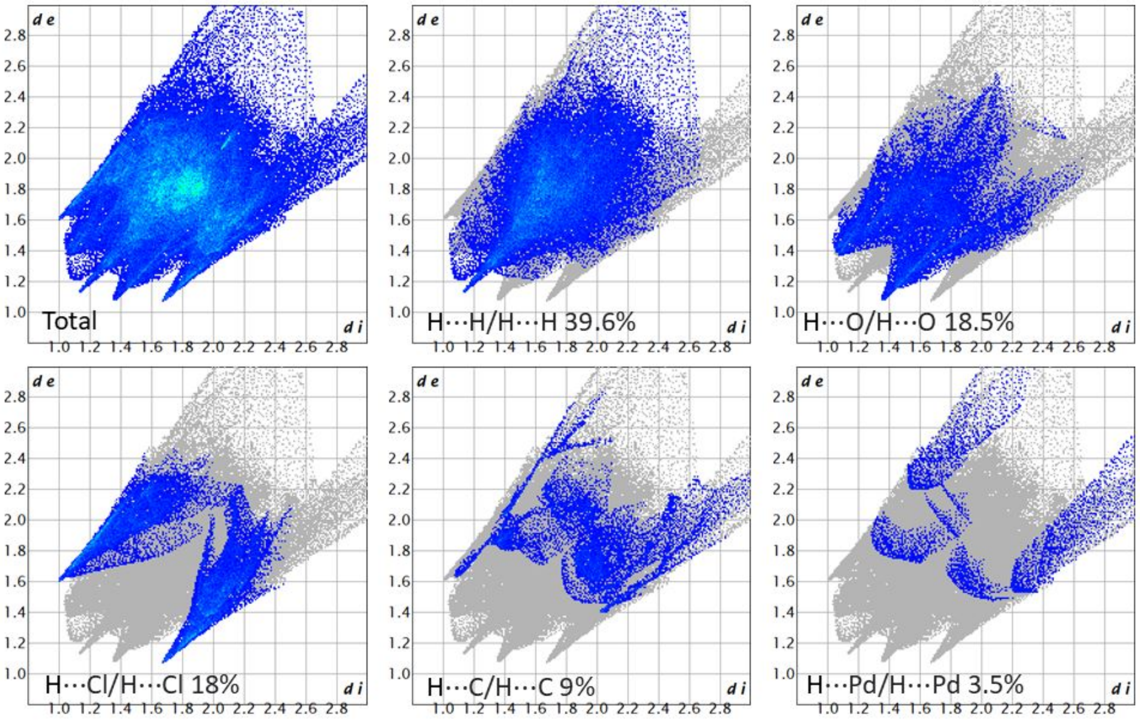

Figure S6. Fingerprint plots for **(4)**, molecule containing Pd1B and PdB.

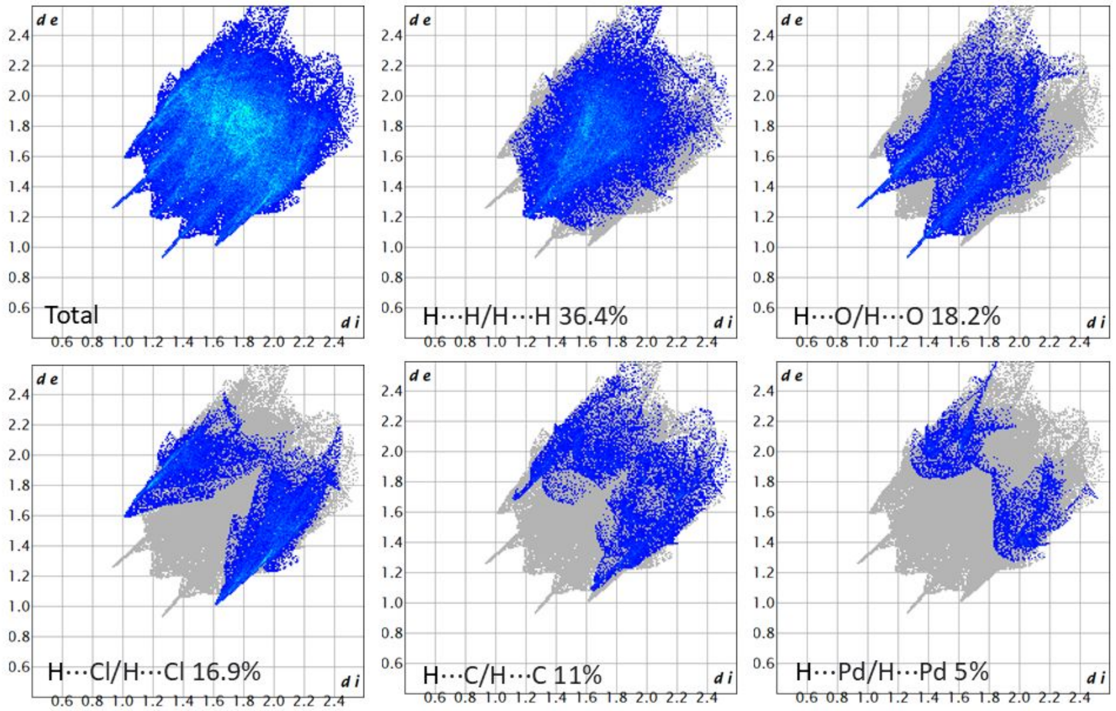

Figure S7. Fingerprint plots for **(4)**, molecule containing Pd1C and Pd1C.

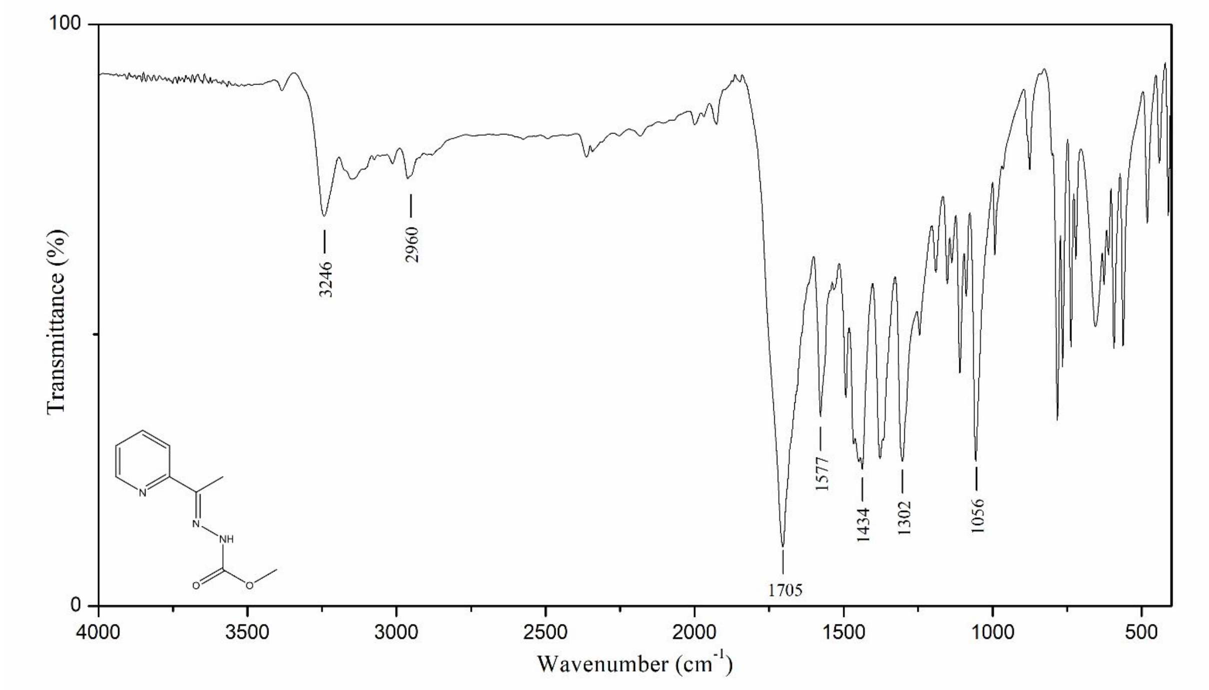

Figure S8. IR spectra of ligand **Hapmc**.

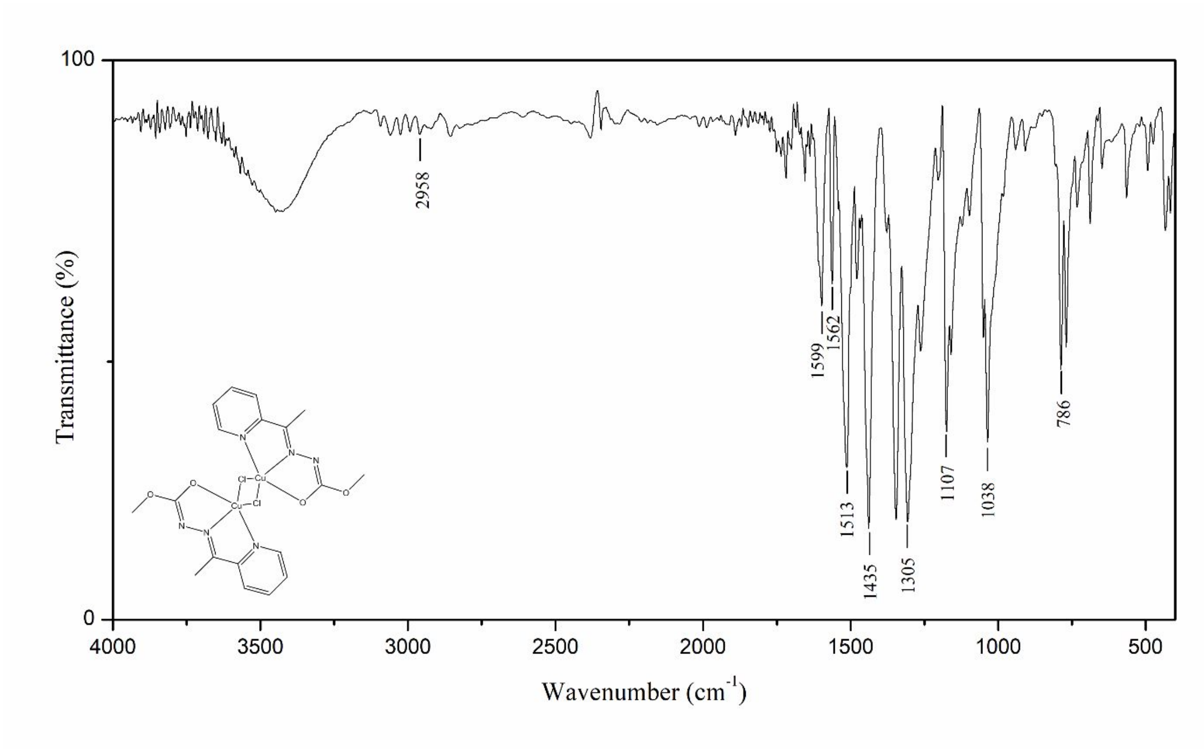

Figure S9. IR spectra of complex **(1)**.

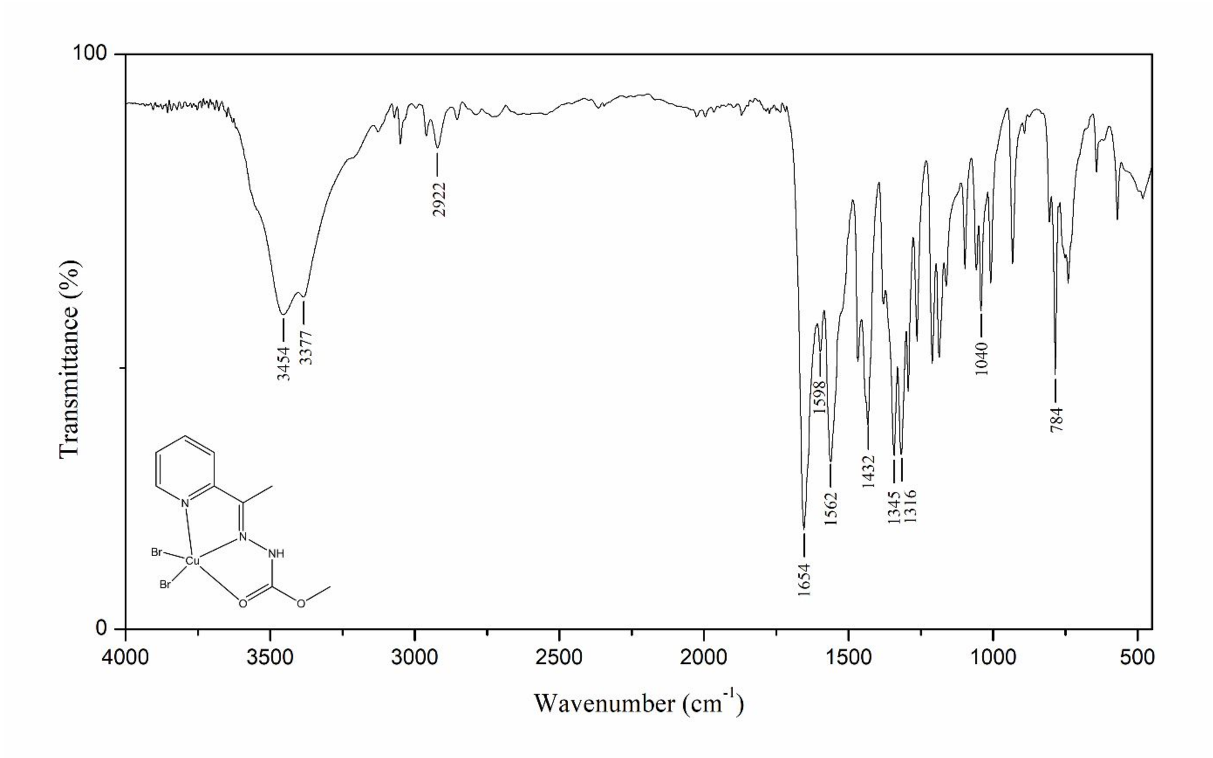

Figure S10. IR spectra of complex **(2)**.

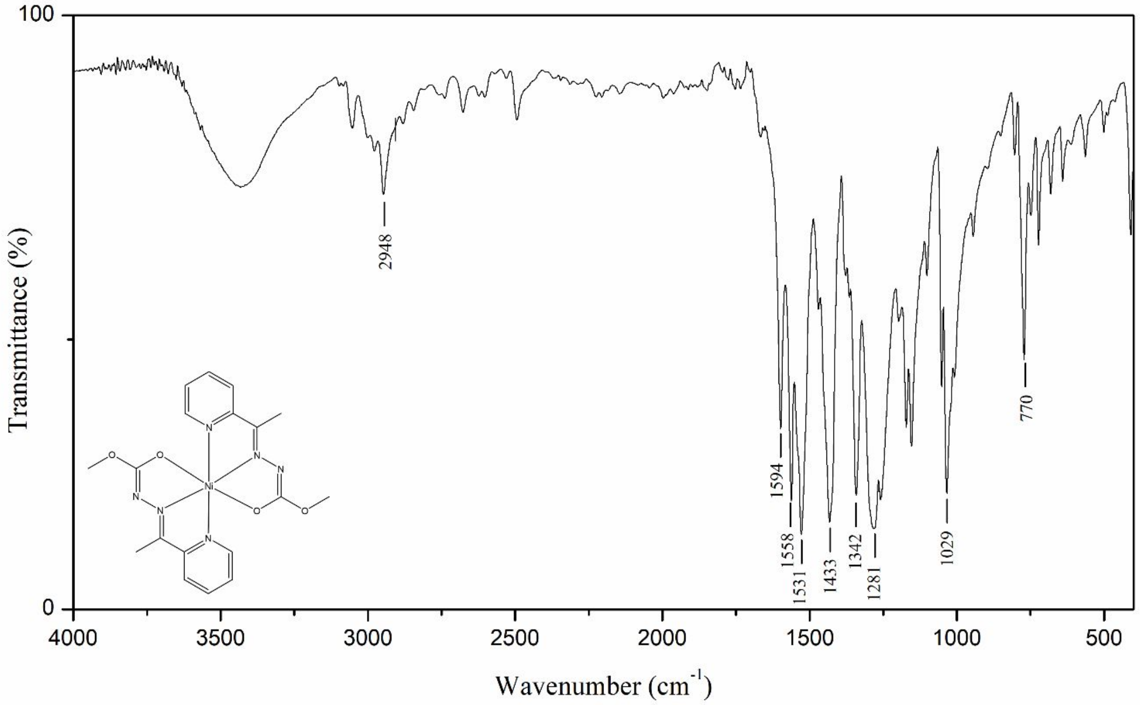

Figure S11. IR spectra of complex **(3)**.

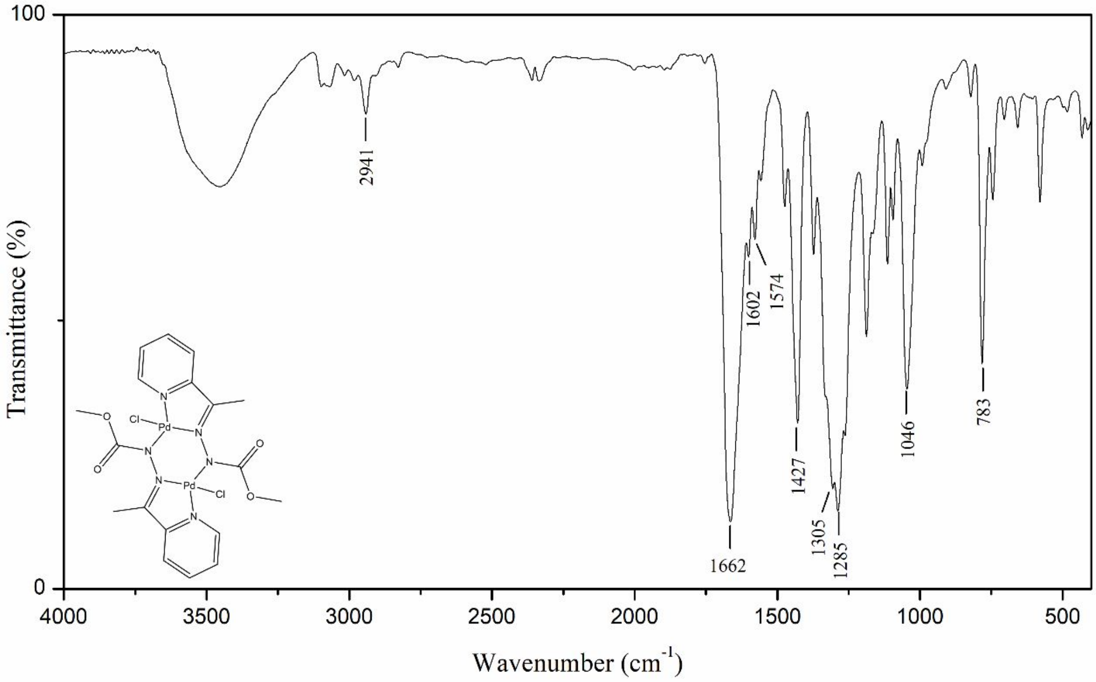

Figure S12. IR spectra of complex **(4)**.

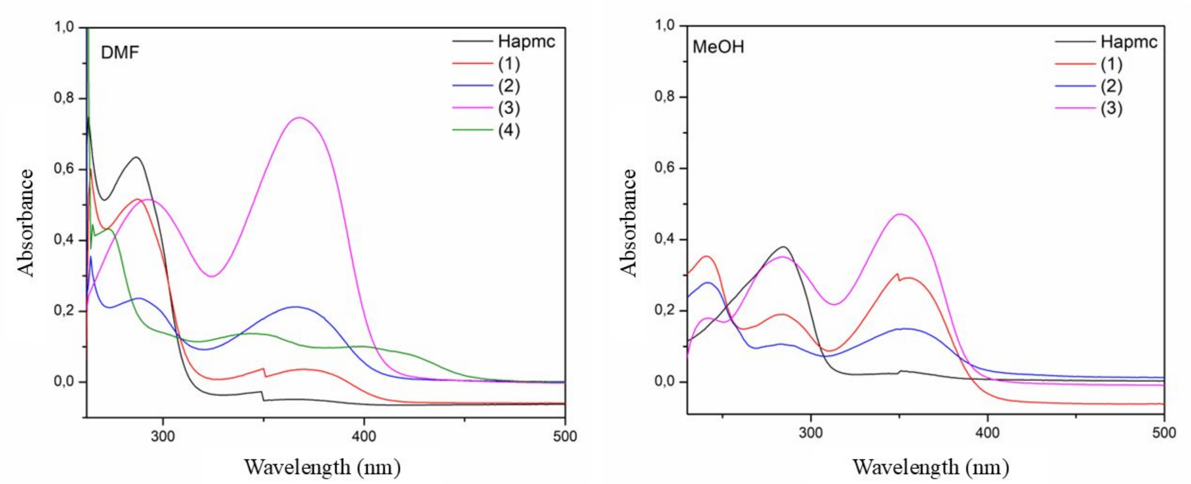

Figure S13. UV-Vis spectra of complexes **(1-4)** and **Hampc**, in DMF and MeOH ($2x10^{-5}$ mol/L).

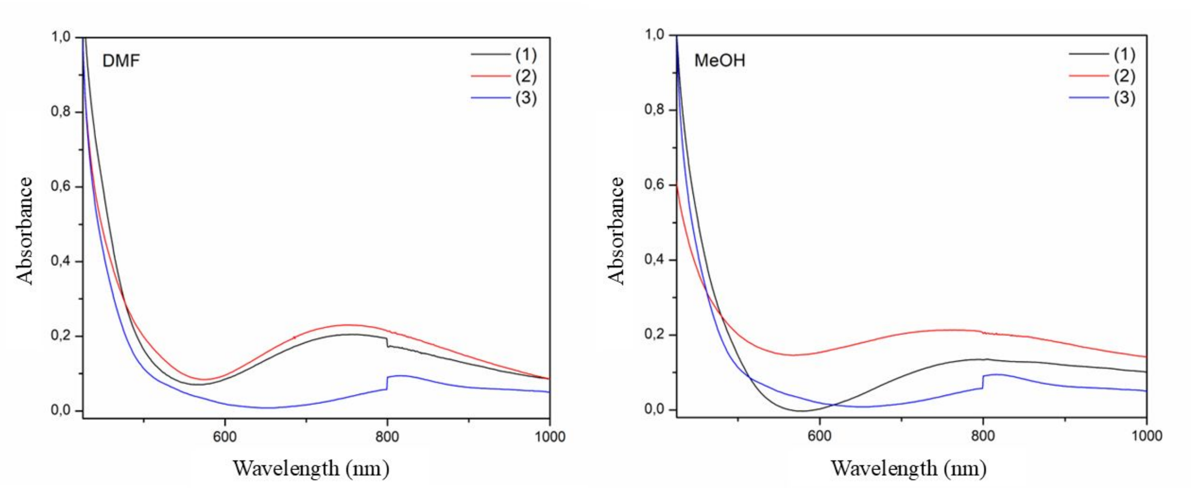

Figure S14. UV-Vis spectra of complexes **(1-4)** and **Hampc**, in DMF and MeOH ($2x10^{-3}$ mol/L).

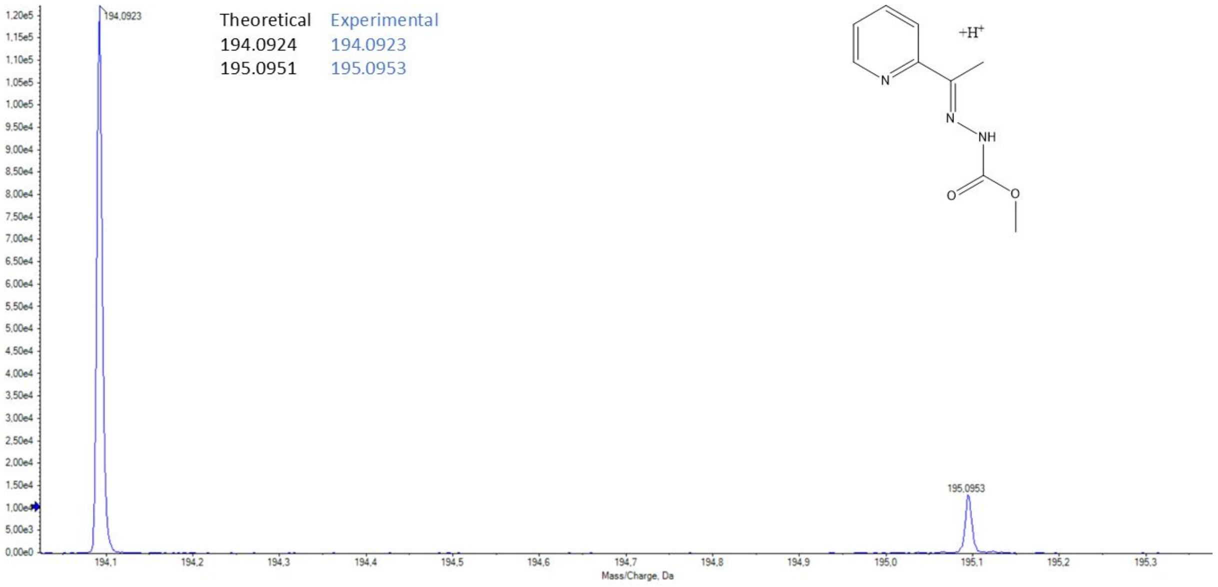

Figure S15. ESI-MS(+) spectra of **Hapmc**.

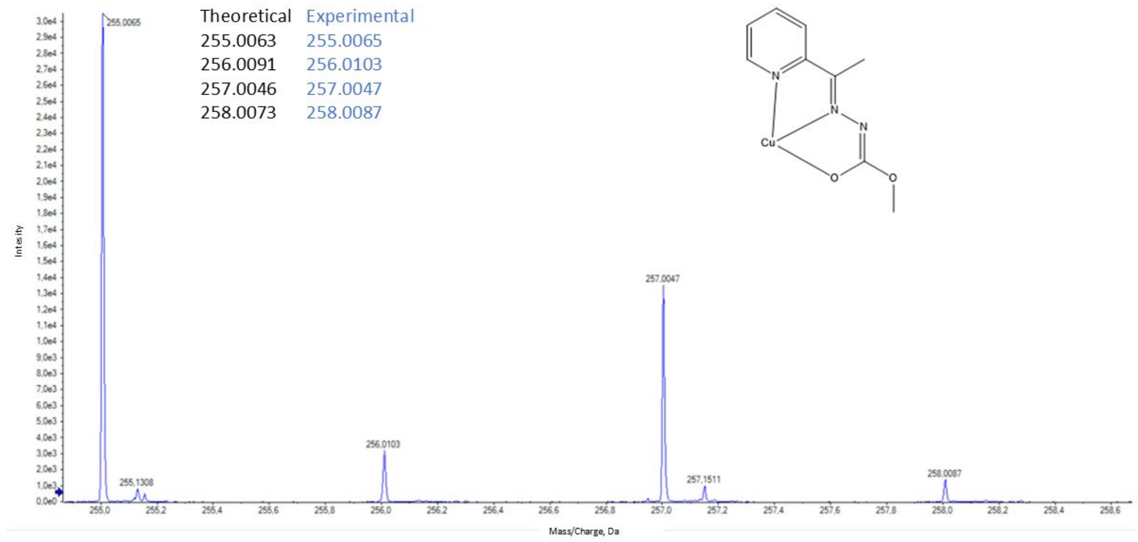

Figure S16. ESI-MS(+) spectra of complex **(1)**.

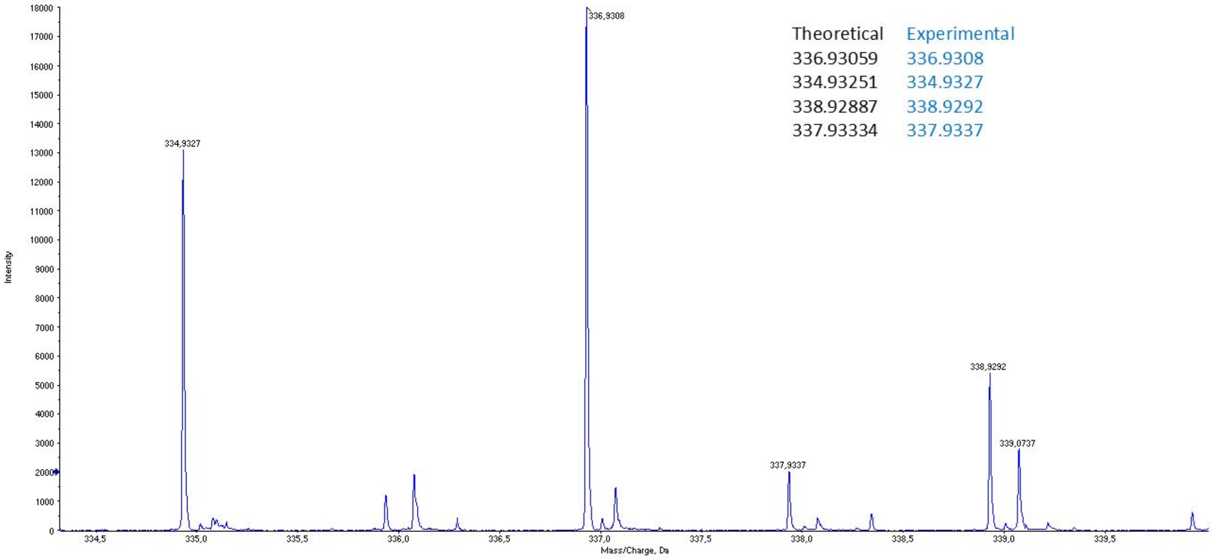

Figure S17. ESI-MS(+) spectra of complex **(2)**.

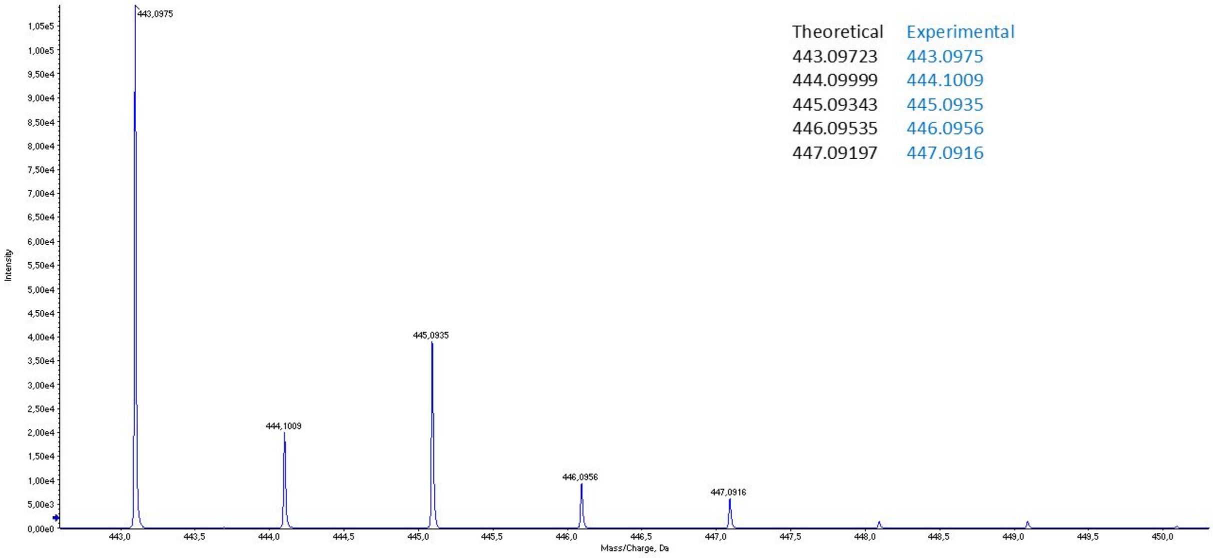

Figure S18. ESI-MS(+) spectra of complex **(3)**.

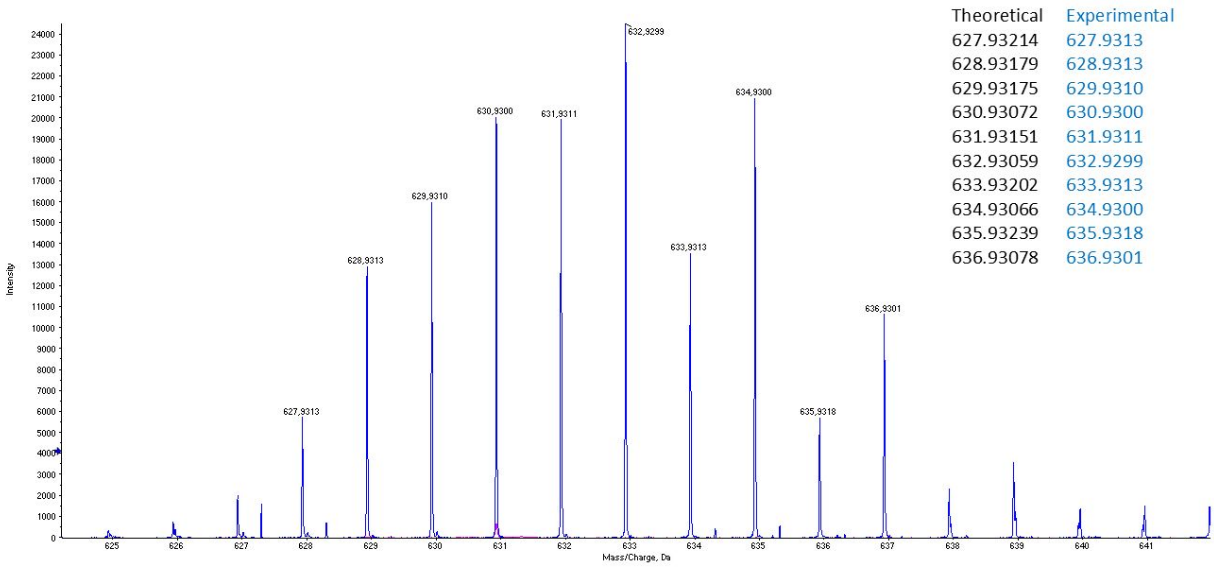

Figure S19. ESI-MSMS(+) spectra of complex **(4)**.

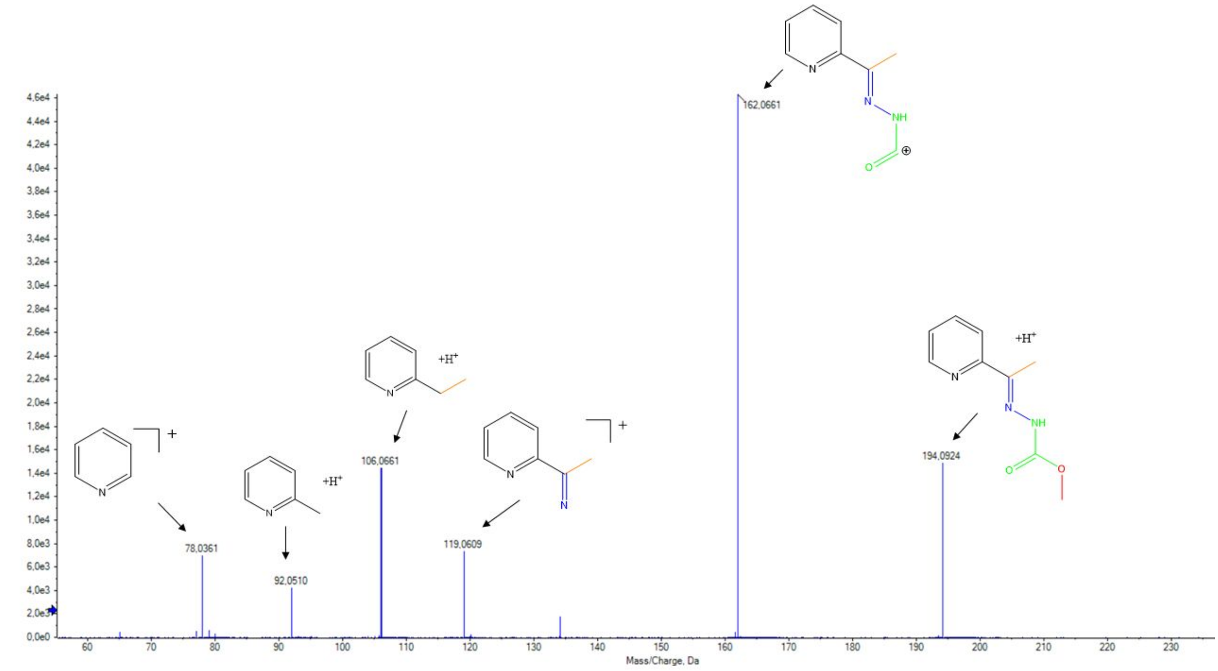

Figure S20. ESI-MSMS(+) spectra of **Hapmc**.

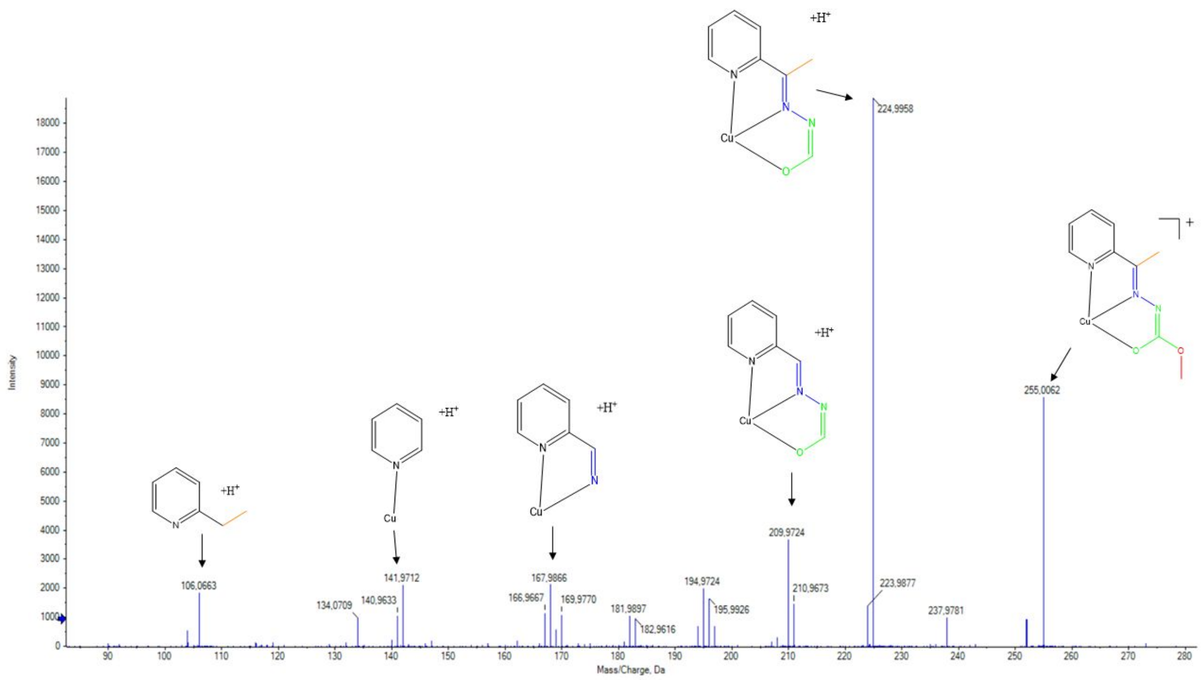

Figure S21. ESI-MSMS(+) spectra of complex **(1)**.

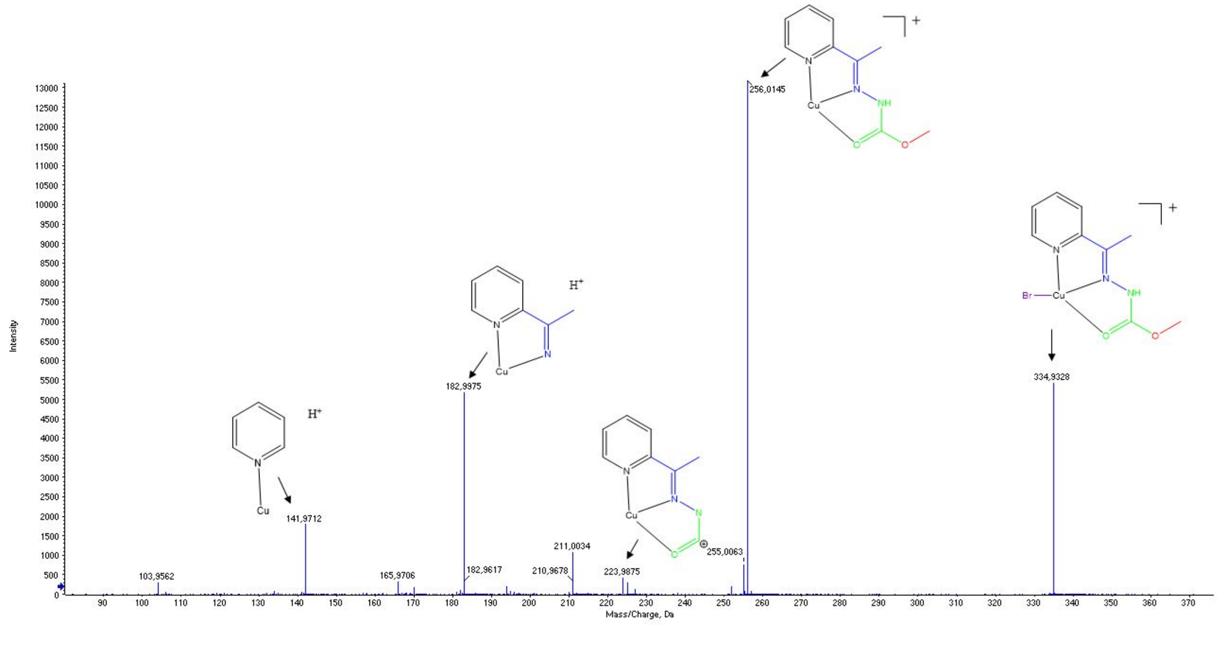

Figure S22. ESI-MSMS(+) spectra of complex **(2)**.

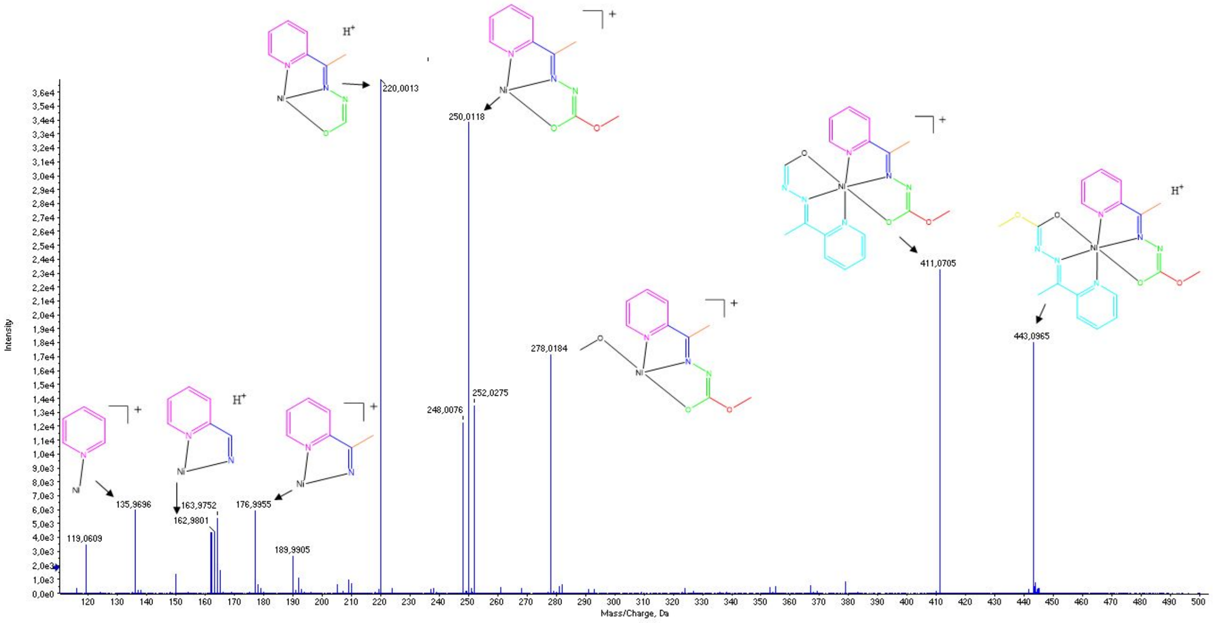

Figure S23. ESI-MSMS(+) spectra of complex **(3)**.

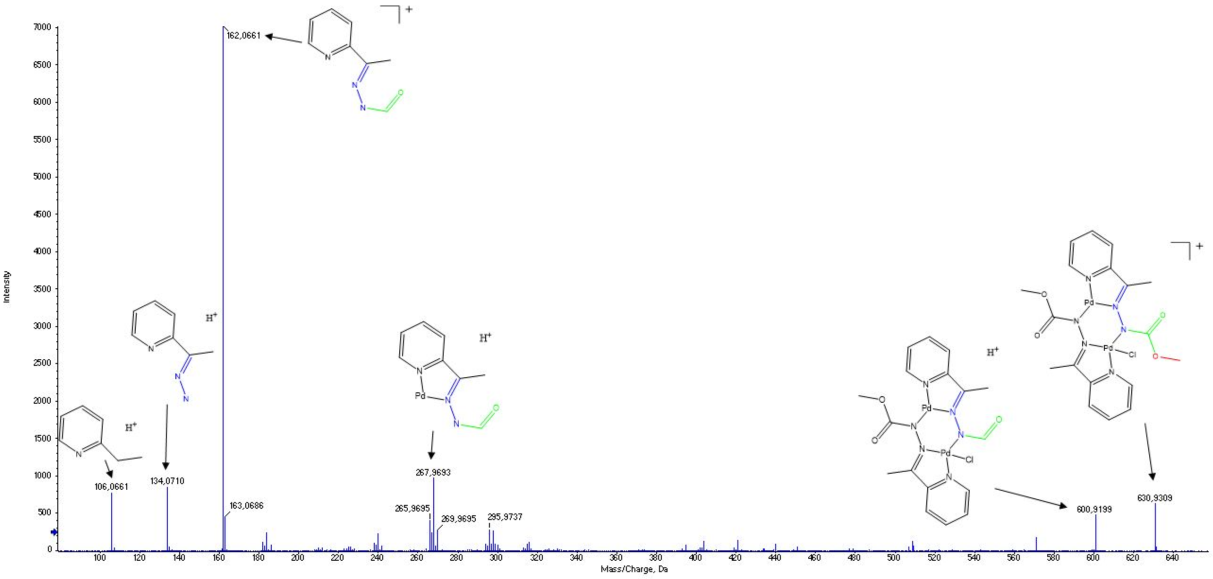

Figure S24. ESI-MSMS(+) spectra of complex **(4)**.

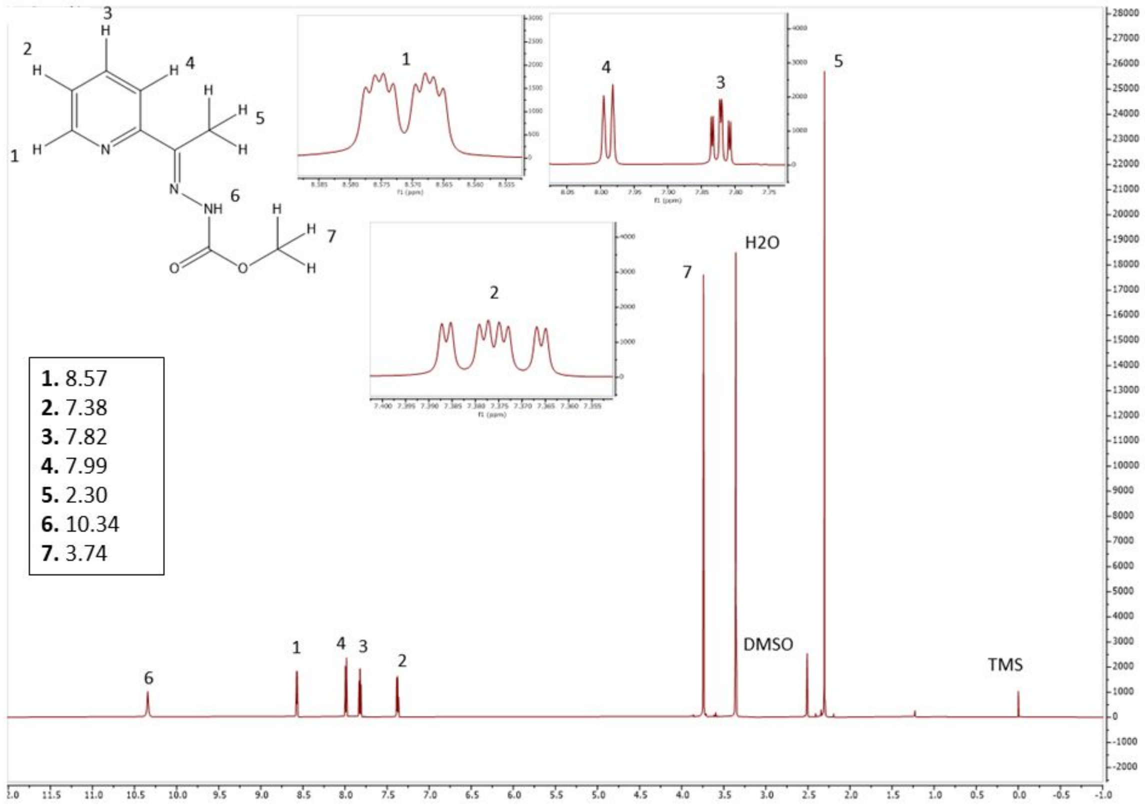

Figure S25. ^{1}H NMR of ligand **Hapmc**.

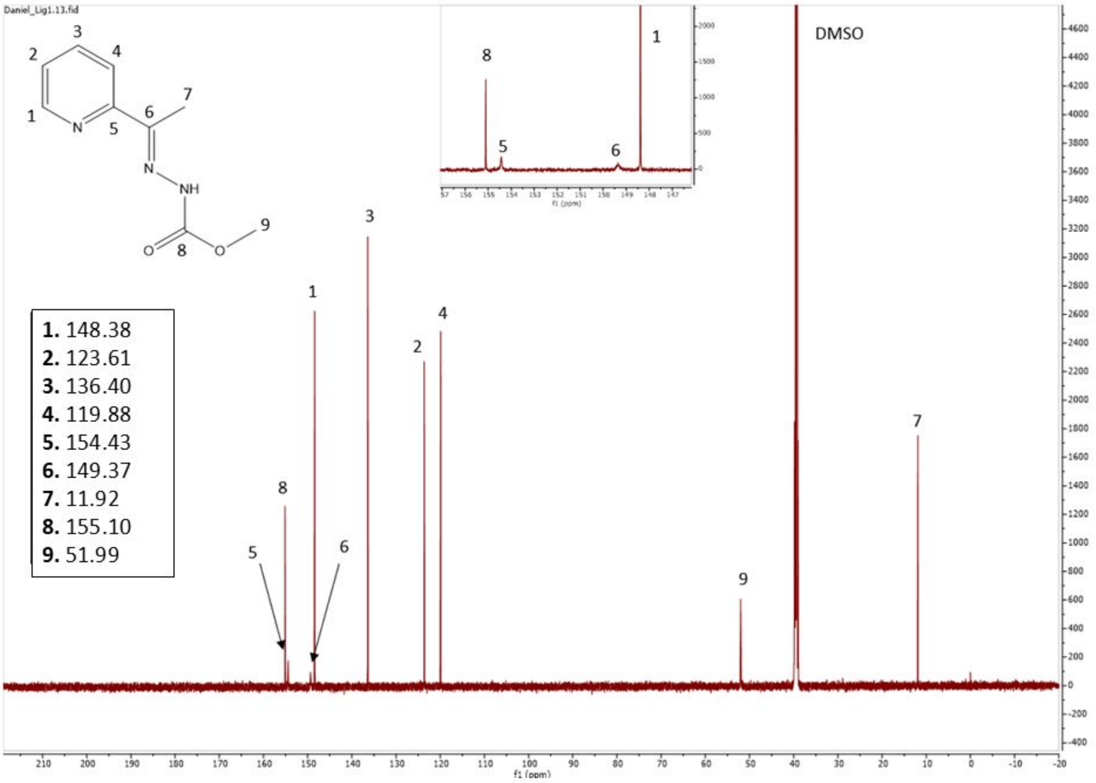

Figure S26. ^{13}C NMR of ligand **Hapmc**.

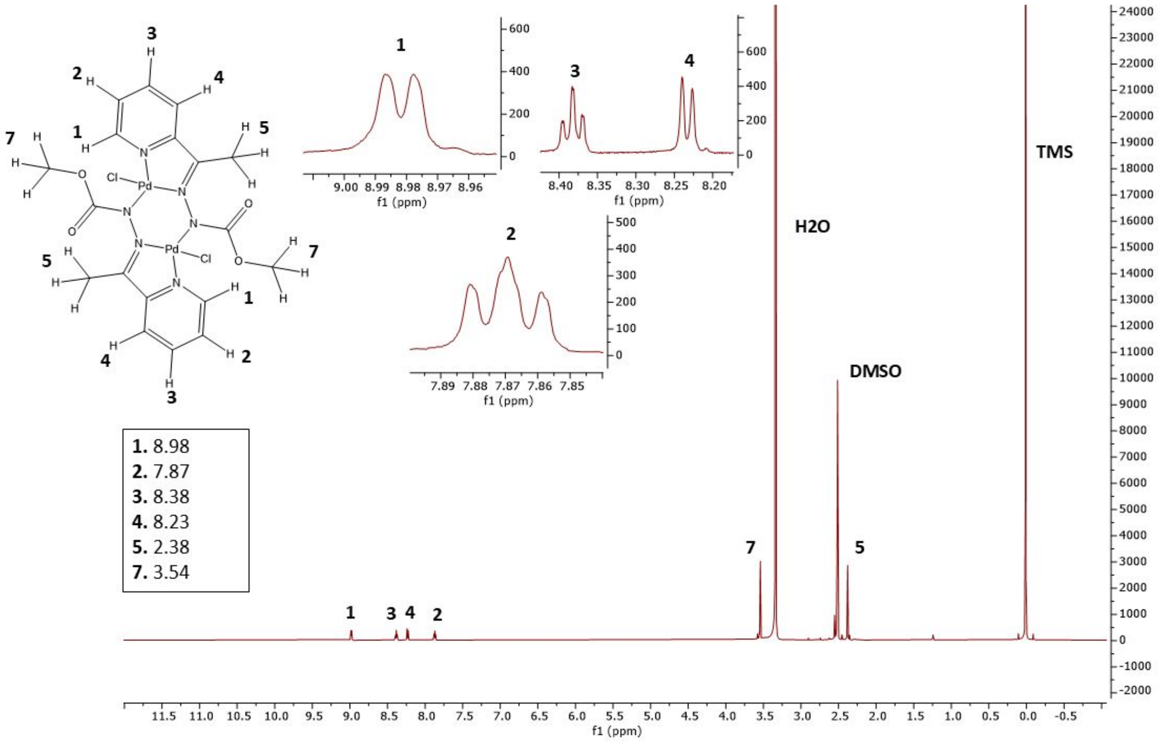

Figure S27. ^{1}H NMR of complex **(4)**.

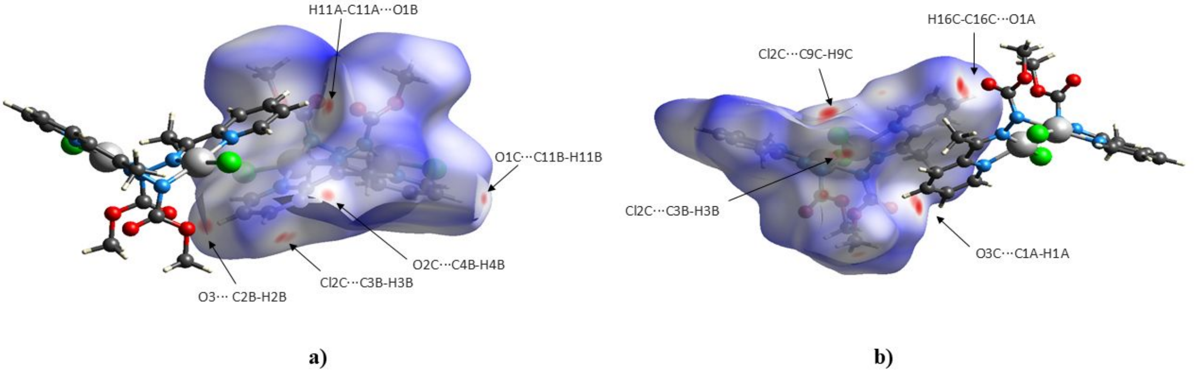

Figure S28. Hirshfeld surface of complex **(4)**. a) molecule containing Pd1B and Pd2B. b) molecule containing Pd1C and Pd2C.

Table S1. Absorption values of **Hapmc** and complexes **(1-4)** with molar absorptivity (logε) in parentheses.

	**$\pi\rightarrow\pi^*$ transition (nm)**		**LMCT transition (nm)**		**d-d transition (nm)**	
	MeOH	DMF	MeOH	DMF	MeOH	DMF
**Hapmc**	283<br>(4.27)	286<br>(4.31)	-	-	-	-
**(1)**	284<br>(3.97)	287<br>(4.25)	315<br>(3.63)	368<br>(3.89)	740<br>(2.02)	749<br>(2.00)
**(2)**	284<br>(3.69)	289<br>(4.04)	301<br>(2.44)	366<br>(2.30)	787<br>(1.81)	749<br>(2.06)
**(3)**	285<br>(4.24)	293<br>(2.65)	319<br>(2.38)	368<br>(2.54)	816<br>(1.65)	810<br>(1.65)
**(4)**	-	272<br>(4.33)	-	342; 400<br>(3.81; 3.69)	-	-

Table S2. Molar conductivity data ($\Omega^{-1}.cm^2.mol^{-1}$) at 0, 24 and 48 hours of DMSO, **Hapmc** and complexes **(1–4)**.

**Compound**	**0 hours**	**24 hours**	**48 hours**
**DMSO**	1.61	1.61	1.61
**Hampc**	1.90	2.37	2.48
**(1)**	58.3	59.6	60.0
**(2)**	32.4	32.6	32.8
**(3)**	2.74	2.74	2.73
**(4)**	3.66	3.62	3.58

Table S3. X-ray diffraction data collection and refinement parameters for **Hapmc** and the complexes **(1–4)**.

	**Hapmc**	**(1)**	**(2)**	**(3)**	**(4)**
Chemical Formula	$C_9H_{11}N_3O_2$	$C_9H_{10}ClN_3O_2Cu$	$C_9H_{13}N_3Br_2O_3Cu$	$C_{18}H_{20}N_6O_4Ni$	$C_{54}H_{60}N_{18}O_{12}Pd_6$
Molecular weight (g.mol^{-1})	193.21	291.190	434.58	443.11	2004.3
Crystal system	Monoclinic	Monoclinic	Triclinic	Monoclinic	Triclinic
Space Group	*P2₁/c*	*C2/c*	*P-1*	*C2/c*	*P-1*
Unit cell					
a (Å)	4.227(7)	15.239(20)	7.567(14)	22.110(3)	7.739(10)
b (Å)	17.696(3)	9.257(12)	8.185(15)	15.185(2)	19.475(3)
c (Å)	12.735(2)	16.800(8)	12.662(2)	15.386(2)	23.551(3)
α (°)	90	90	80.905(4)	90	101.746
β (°)	92.289(4)	112.160(4)	94.435(7)	120.813(3)	96.047(3)
γ (°)	90	90	66.070(3)	90	91.011
V (Å^3)	951.7(3)	2194(5)	707,8(2)	4436.4(12)	3453.3(8)
Z	4	8	2	8	2
Density (mg.cm^{-3})	1.348	1.763	2.039	1.327	1.928
Absorption coefficient μ (mm^{-1})	0.089	2.221	7.911	0.908	1.832
2θ range for data collection (º)	1.97 – 25.38	5.238 – 50.69	3.25 – 50.86	3.434 – 50.948	2.138 – 50.732
Index range h, k, l	$-5 \leq h \leq 5$	$-18 \leq h \leq 18$	$-9 \leq h \leq 9$	$-26 \leq h \leq 26$	$-9 \leq h \leq 9$
	$-21 \leq k \leq 21$	$-11 \leq k \leq 11$	$-9 \leq k \leq 9$	$-18 \leq k \leq 18$	$-23 \leq k \leq 23$
	$-15 \leq l \leq 15$	$-20 \leq l \leq 20$	$-15 \leq l \leq 15$	$-18 \leq l \leq 18$	$-28 \leq l \leq 28$
Reflections collected	9207	10406	16333	28855	45203
Independent reflections /R(int)	1735/0.044	1986/0.103	2603/0.085	4121/0.073	12662/0.097
Absorption correction	Multi-scan	Multi-scan	Multi-scan	Multi-scan	Multi-scan
Max/min transmission	0.745/0.67	0.677/0.495	0.745/0.276	0.842/0.681	0.745/0.616
F(000)	408	1176	422	1840	1968
Goodness-of-fit (F^2)	1.039	1.021	1.039	1.037	1.008
Final R indexes [I>=2σ(I)]	$R_1 = 0.041$	$R_1 = 0.047$	$R_1 = 0.038$	$R_1 = 0.039$	$R_1 = 0.057$
	$wR_2 = 0.111$	$wR_2 = 0.097$	$wR_2 = 0.099$	$wR_2 = 0.102$	$wR_2 = 0.135$
Largest diff. peak/hole (e.Å^{-3})	0.17/-0.13	0.53/-0.52	0.76/-0.77	0.23/-0.31	0.85 /-0.95
CCDC	2420651	2420652	2420653	2420654	2420655
